# Supplementary material for: A novel anti-B7-H3 chimeric antigen receptor from a single-chain antibody library for immunotherapy of solid cancers
Source: Mol Ther Oncolytics. 2022 Aug 25;26:429–43. doi: 10.1016/j.omto.2022.08.008 (PMC9467911; doi:10.1016/j.omto.2022.08.008)
Supplement: Document S1. Figures S1–S9 [file mmc1.pdf]

OMTO, Volume *26*

Supplemental information

A novel anti-B7-H3 chimeric antigen receptor from a single-chain antibody library for immunotherapy of solid cancers

Kathleen Birley, Clara Leboreiro-Babe, Enrique Miranda Rota, Magdalena Buschhaus, Artemis Gavriil, Alice Vitali, Maria Alonso-Ferrero, Lee Hopwood, Lara Parienti, Gabrielle Ferry, Barry Flutter, Nourredine Himoudi, Kerry Chester, and John Anderson

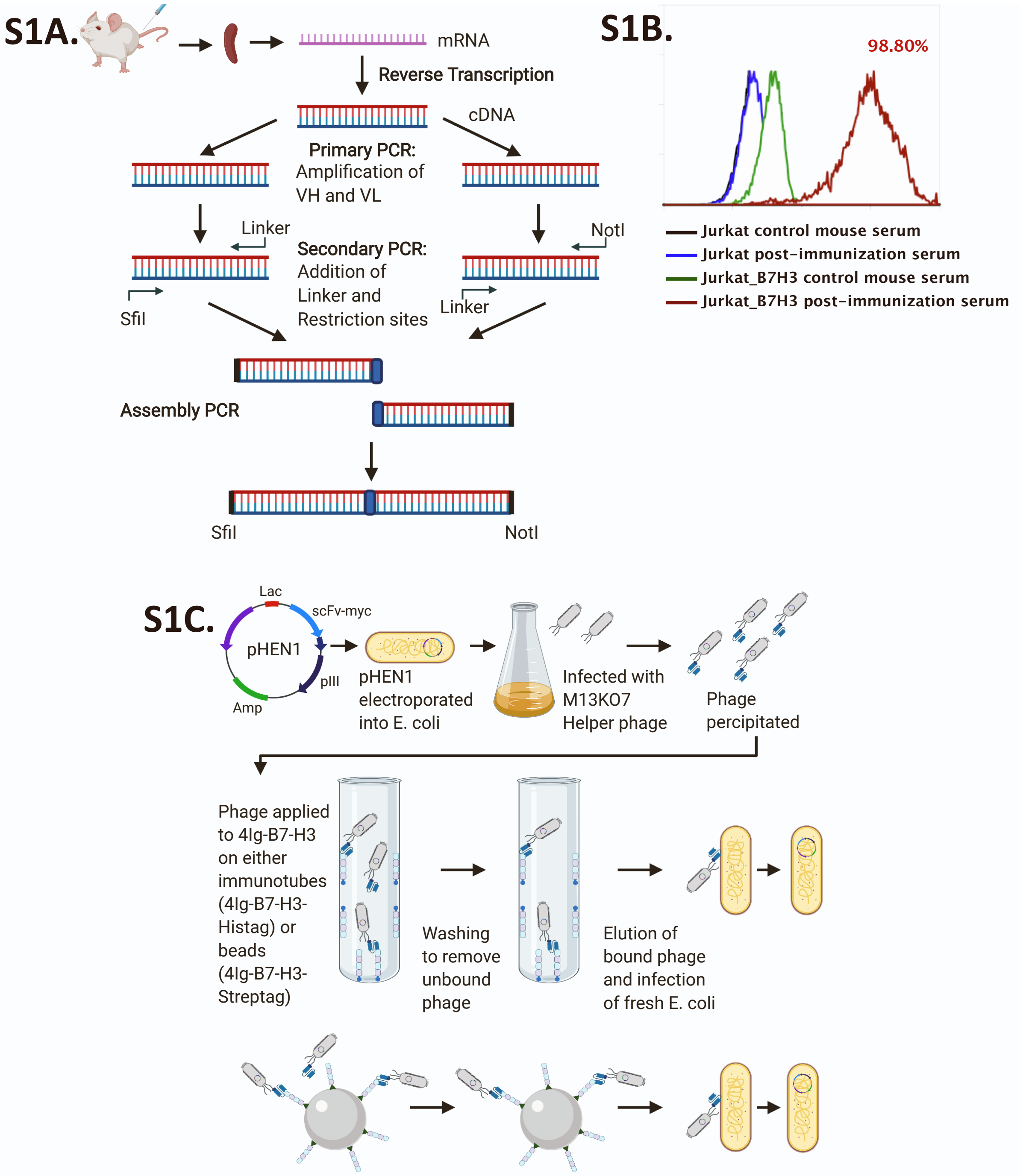

S1D.

	Immunized Library	
**Library Size**	$2.06x10^7$	
	**Immunotube panning**	**Bead panning**
**Library Size**	$1.20x10^4$	$1.05x10^4$

Figure S1. *Production and panning of the anti-B7-H3 library*. **A.** Production of the phage display library. Mice were immunized with a B7-H3-mouseFc fusion protein. The spleens were collected from the immunized mouse, mRNA extracted reverse transcribed into corresponding VH and VL cDNA. PCR reactions were used to add a linker and myc-tag before the scFv-myc was cloned into the pHEN phagemid. **B.** Testing of the immunized mouse serum against B7-H3 +/- Jurkat cells. **C.** The phage display library was panned against 4Ig-B7-H3 immobilized on immunotubes or magnetic beads. **D.** The size of the immunized library and the immunotube and magnetic bead panned libraries as estimated by serial dilution of phage-infected E. *coli*.

S2A.

	BB5	TB8	BH6	TE9	BF9	BB1	TA12	BF12	TA6	BC10	BA5	BF2	BG5	BG4	BD9	TC6	TF9
BB5	100.00	68.05	79.52	78.16	78.54	75.91	78.26	79.27	80.15	78.37	79.21	78.16	79.42	78.91	78.54	78.16	78.41
TB8	68.05	100.00	78.91	81.31	80.93	78.81	80.93	81.69	80.18	80.05	82.78	80.71	82.60	82.60	80.83	81.34	82.09
BH6	79.52	78.91	100.00	92.37	92.11	90.60	92.88	91.60	92.97	92.83	93.11	91.99	91.23	92.88	91.99	91.49	92.25
TE9	78.16	81.31	92.37	100.00	96.34	91.55	93.94	94.19	91.98	93.13	94.17	93.94	94.19	94.95	95.33	93.56	94.57
BF9	78.54	80.93	92.11	96.34	100.00	91.80	93.81	95.45	92.88	93.51	94.55	93.94	94.44	94.57	96.46	94.57	95.45
BB1	75.91	78.81	90.60	91.55	91.80	100.00	95.71	93.32	90.47	90.85	90.89	91.17	90.67	91.55	90.92	91.30	91.05
TA12	78.26	80.93	92.88	93.94	93.81	95.71	100.00	95.59	92.62	93.26	92.78	93.06	92.93	93.31	92.42	92.93	92.55
BF12	79.27	81.69	91.60	94.19	95.45	93.32	95.59	100.00	92.49	92.24	94.80	93.56	94.82	94.57	94.19	95.45	94.82
TA6	80.15	80.18	92.97	91.98	92.88	90.47	92.62	92.49	100.00	93.47	94.26	92.25	92.88	93.01	92.88	92.63	93.52
BC10	78.37	80.05	92.83	93.13	93.51	90.85	93.26	92.24	93.47	100.00	92.88	93.52	93.39	93.27	94.54	93.52	94.03
BA5	79.21	82.78	93.11	94.17	94.55	90.89	92.78	94.80	94.26	92.88	100.00	93.67	94.68	95.32	94.56	94.43	95.95
BF2	78.16	80.71	91.99	93.94	93.94	91.17	93.06	93.56	92.25	93.52	93.67	100.00	95.84	95.84	96.09	96.09	96.47
BG5	79.42	82.60	91.23	94.19	94.44	90.67	92.93	94.82	92.88	93.39	94.68	95.84	100.00	96.47	96.47	96.85	96.97
BG4	78.91	82.60	92.88	94.95	94.57	91.55	93.31	94.57	93.01	93.27	95.32	95.84	96.47	100.00	95.59	96.09	97.98
BD9	78.54	80.83	91.99	95.33	96.46	90.92	92.42	94.19	92.88	94.54	94.56	96.09	96.47	95.59	100.00	97.60	97.10
TC6	78.16	81.34	91.49	93.56	94.57	91.30	92.93	95.45	92.63	93.52	94.43	96.09	96.85	96.09	97.60	100.00	97.86
TF9	78.41	82.09	92.25	94.57	95.45	91.05	92.55	94.82	93.52	94.03	95.95	96.47	96.97	97.98	97.10	97.86	100.00

>60% Identical
>70% Identical
>80% Identical
>90% Identical
>95% Identical
100% Identical

S2B.

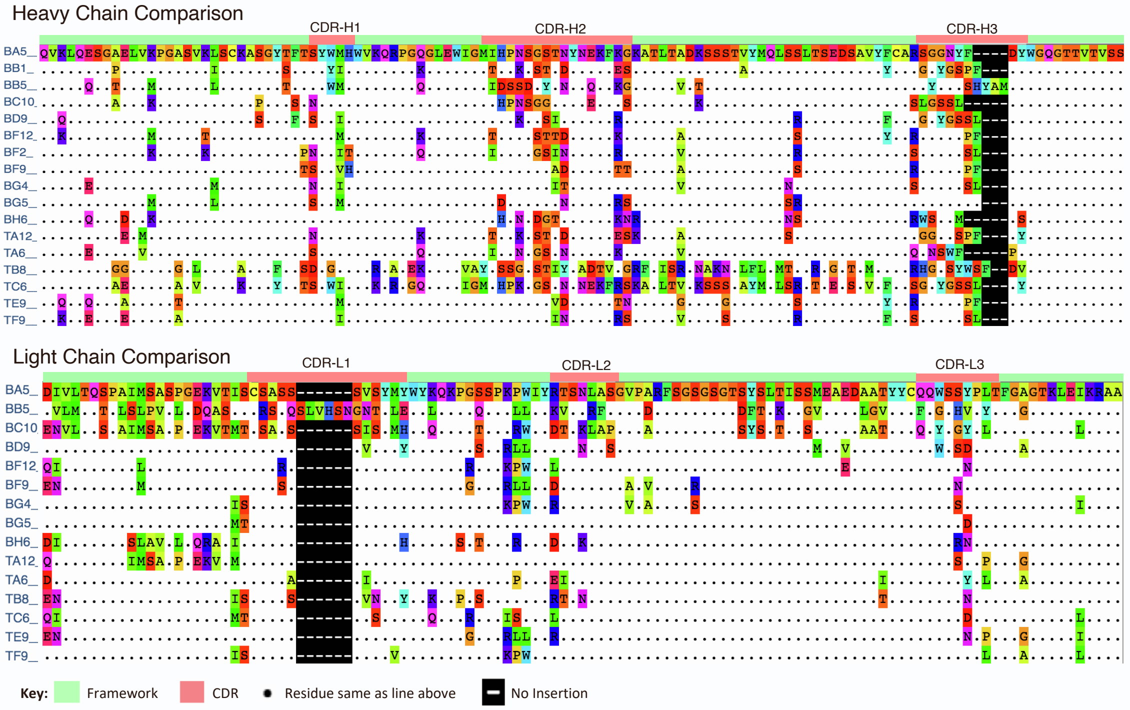

Figure S2. *The diversity of the anti-B7-H3 scFv*. **A.** A heat map showing the percentage similarities between the 17 different anti-B7-H3 scFv **B.** Alignment of the anti-B7-H3 scFv heavy and light chains arranged in alphabetical order as analyzed by Abysis (http://www.abysis.org/abysis/)

S3A.

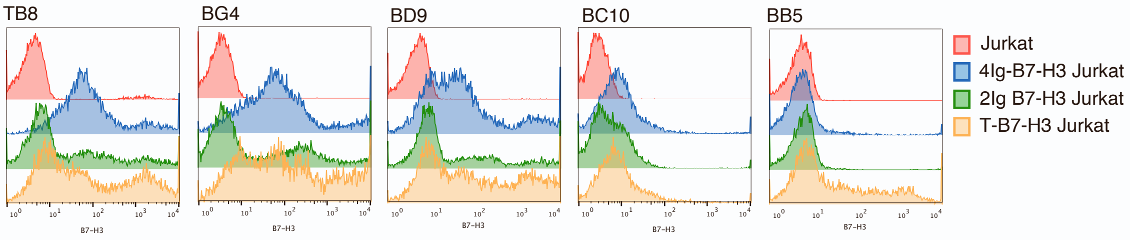

S3B.

CAR	Range	Average
TC6	61.3% - 84.1%	71.9%
TE9	58.1% - 90.5%	76.3%
TF9	34.7% - 81.9%	64.4%
BF9	72.2% - 79.6%	74.8%
BH6	40.3% - 82.1%	58.2%
GD2	23.3% - 81.3%	45.1%

S3C.

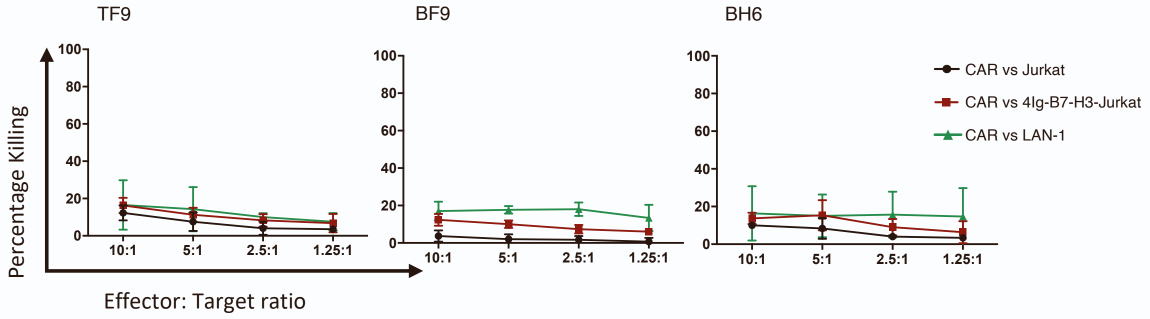

Figure S3. *The scFv not taken forward for further evaluation in CAR T cells*. **A.** The binding of scFv-Fc not taken forward against different cell-bound isoforms of B7-H3. Representative 1 of 2. **B.** The range and average transduction efficiency as determined on flow cytometry **C.** Cr^{51} cytotoxicity assay of CAR-T cells not taken forward against isogenic B7-H3 +/- cell lines and LAN-1 cells (mean and SD, TF9 n = 4, BF9 and BH6 n = 3).

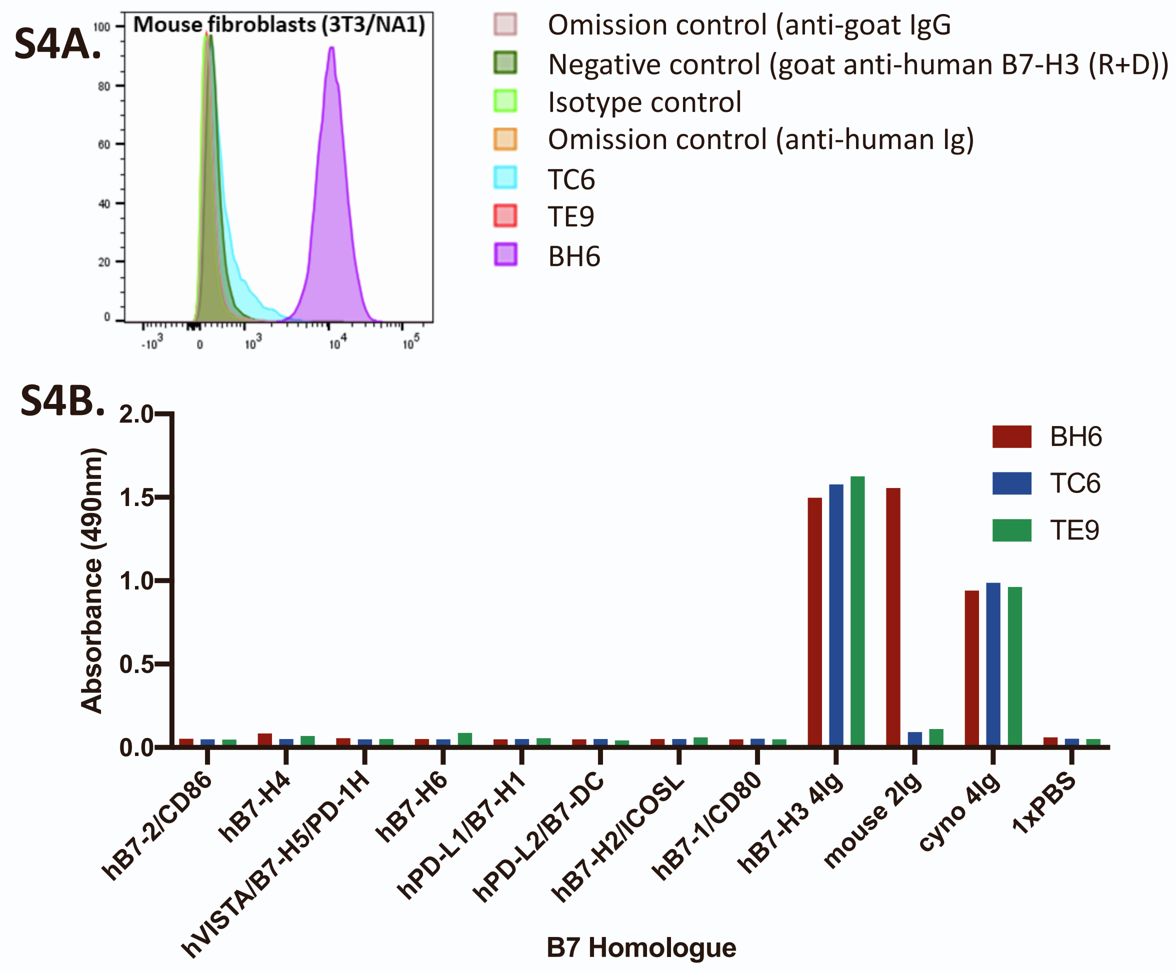

Figure S4. *Specificity of TE9, TC6 and BH6*. **A.** Staining of mouse 3T3/NA1 with TE9, TC6 and BH6 antibodies **B.** Results of ELISA showing binding of antibodies TE9, TC6 and BH6 against recombinant human B7 family proteins and non-human primate B7-H3

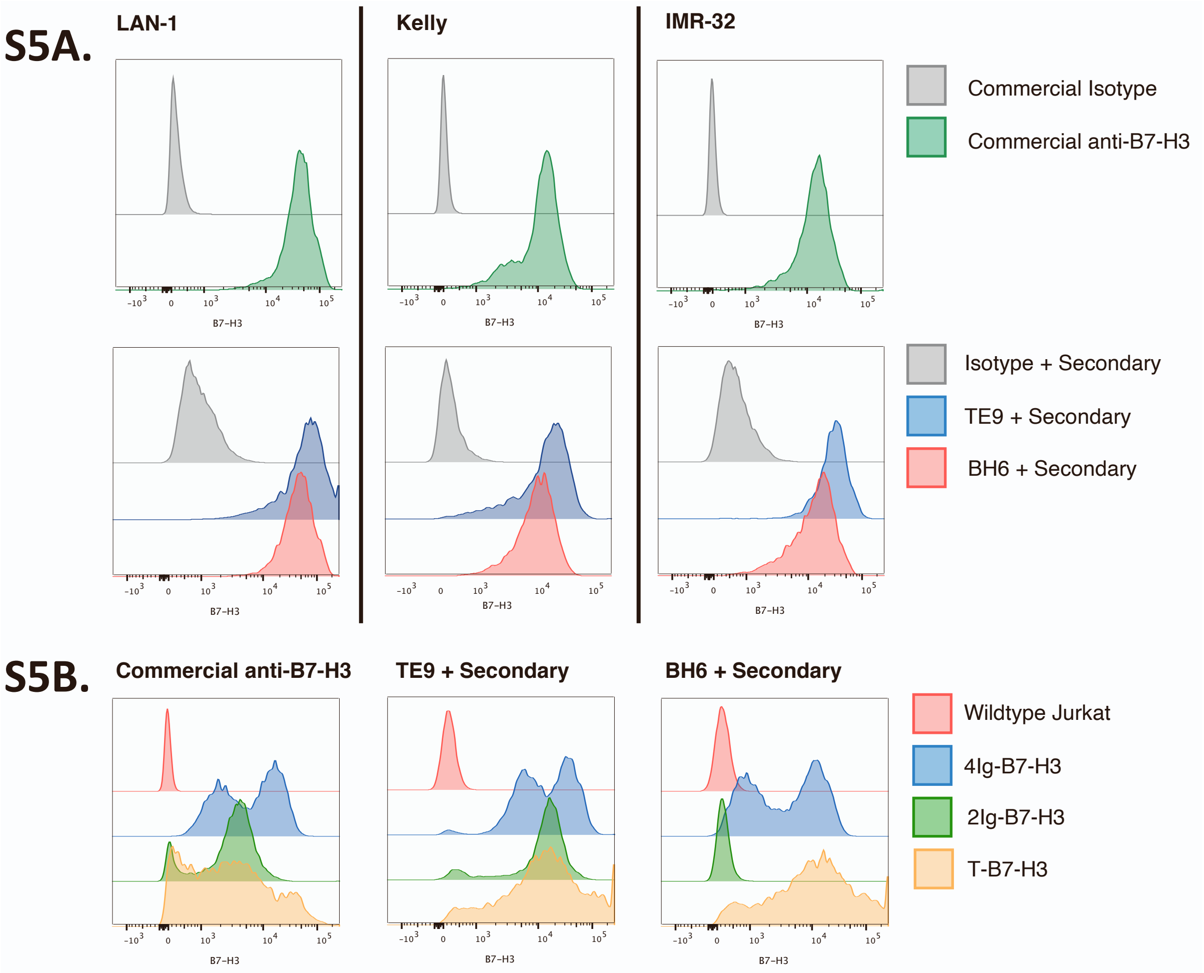

Figure S5. *Chimeric antibodies of TE9 and BH6 show a similar pattern of staining as scFv-Fc proteins and stain neuroblastoma cell lines*. **A.** Chimeric antibodies of TE9 and BH6 were used to stain 3 neuroblastoma cell lines, LAN-1, Kelly and IMR-32. The top line shows staining with a commercial directly conjugated antibody and the bottom line with the chimeric antibodies and an anti-human secondary. **B.** Commercial anti-B7-H3 antibodies and the chimeric antibodies TE9 and BH6 were used to stain the 3 B7-H3 positive Jurkat cell lines, 4Ig-B7-H3, 2Ig-B7-H3 and T-B7-H3

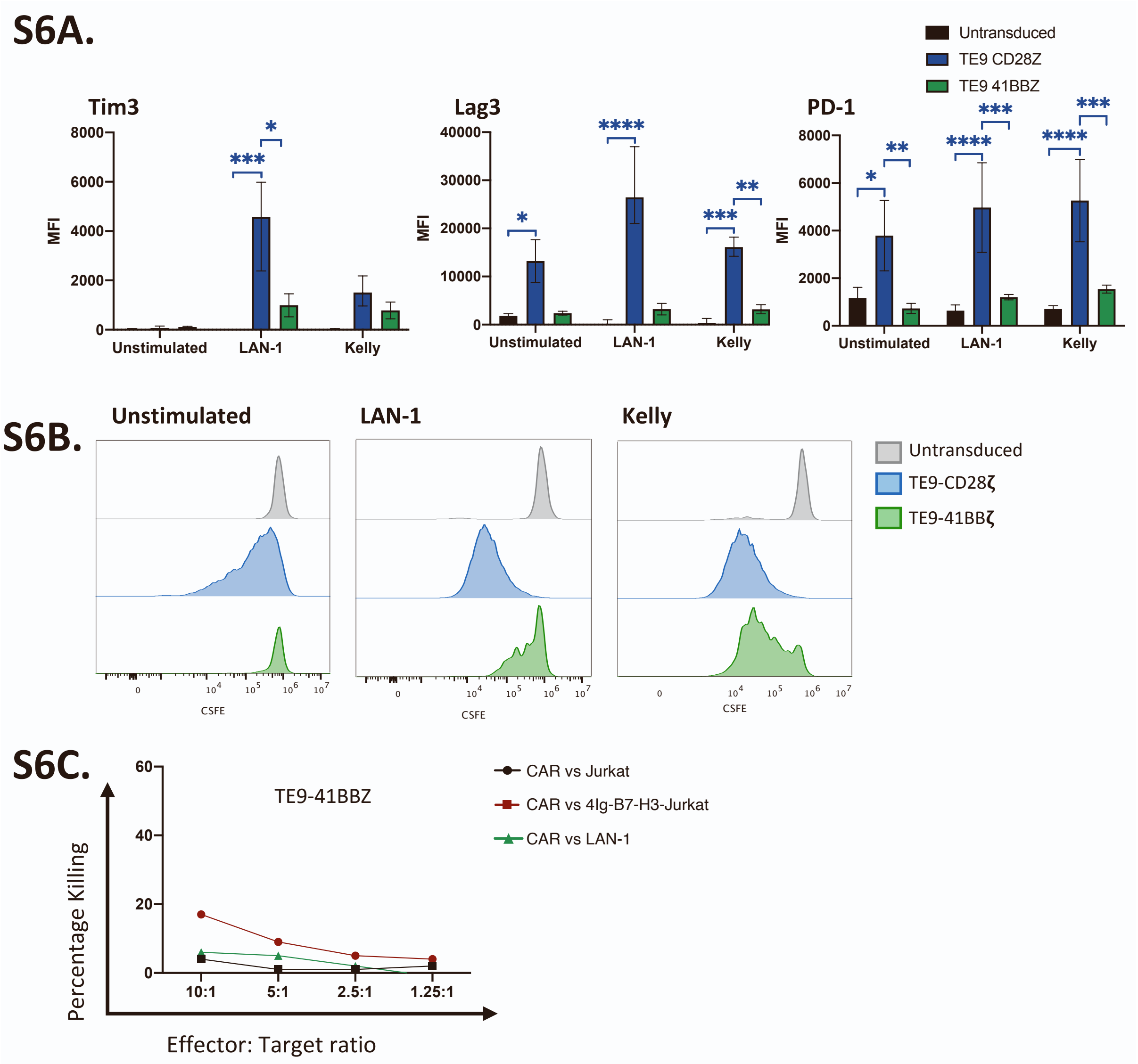

Figure S6. *TE9-28ζ expresses higher levels of Tim3, Lag3 and PD-1 following 7-day co-culture with antigen positive target cells than TE9-41BBζ.* **A.** CAR-T cells and Untransduced T-cells were cultured with LAN-1 or Kelly targets, or no antigen stimulus for 7 days then restimulated with fresh antigen targets and incubated for a further 24 hours. Cells were stained for Tim3, Lag3 and PD-1 (mean and range, n = 3, * $p \leq 0.05$; ** $p \leq 0.01$; *** $p \leq 0.001$; **** $p<0.0001$) **B.** Proliferation of TE9-CD28ζ, TE9-41BBζ and untransduced T-Cells when following 7-day co-culture and repeat stimulation assay with either LAN-1, Kelly or UT as measured using CSFE dilution, n = 1. **C.** Cytotoxicity of TE9-41BBζ against B7-H3 +/- Jurkat cells and LAN-1 measured using a chromium release assay, n = 1.

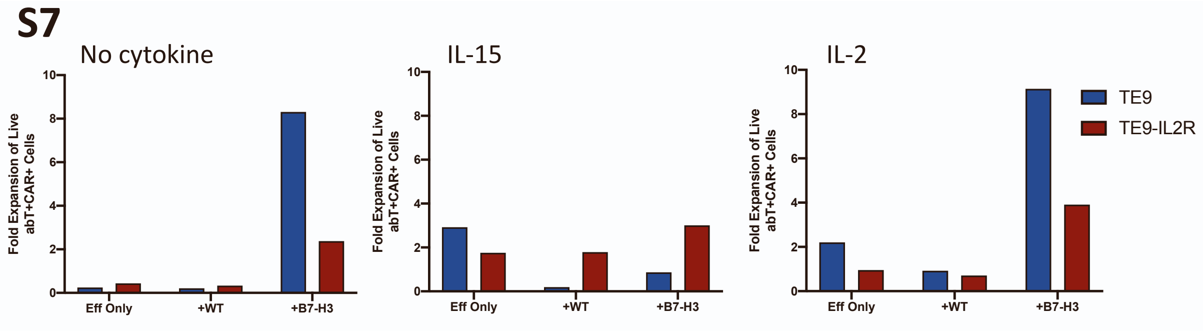

Figure S7. *TE9-28-ILR2ζ does not show proliferative superiority compared with TE9-28ζ.* **A.** CAR T cells transduced with either TE9-28ζ or TE9-28-ILR2ζ and cultured with B7-H3+/- isogenic Jurkat cell lines. Cells were cultured in the presence of IL-15, IL-2, or no cytokine stimulus. Proliferation in the form of fold change was measured on day 6 of culture using precision count beads (n = 1)

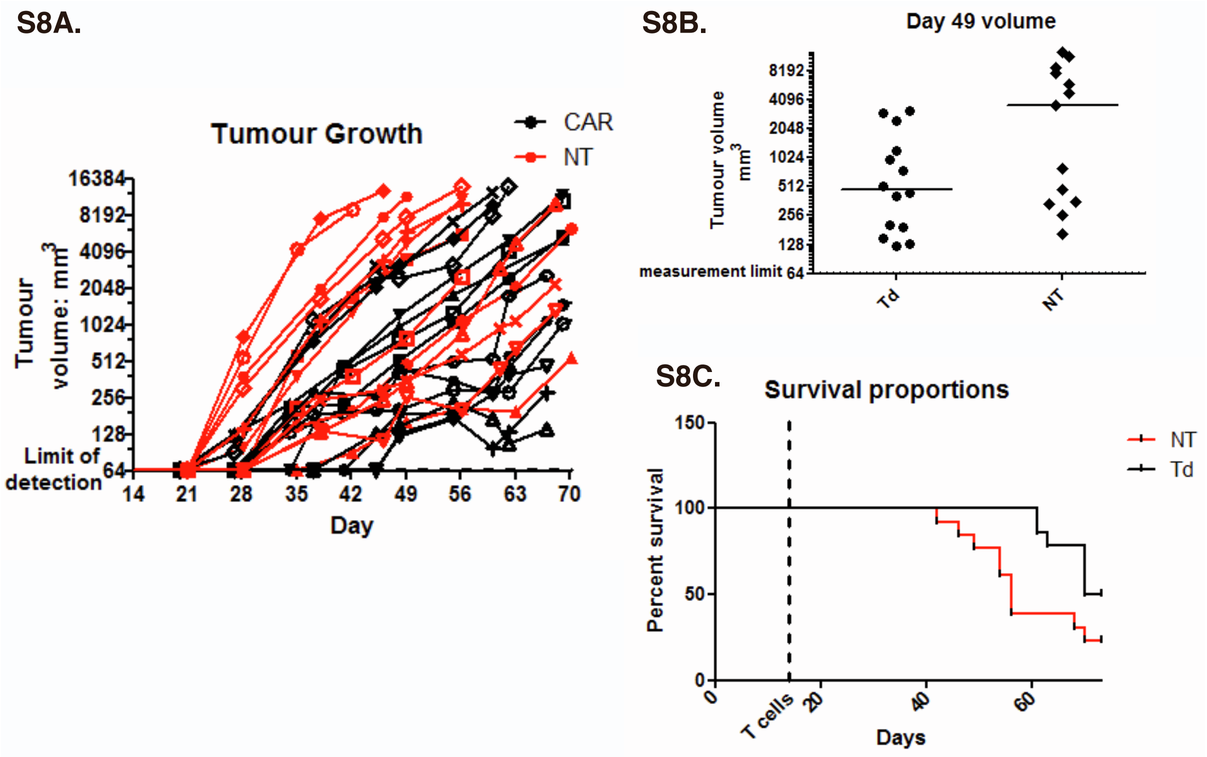

Figure S8 *Anti-GD2-CD28-CD3ζ CAR T cells do not significantly increase survival or reduce tumor size in NSG mice bearing LAN-1 tumors*. 1x10^6 LAN-1 cells in Matrigel were injected into the flank of NSG mice. When the majority of the tumors reached threshold for detection (64mm^3) mice were treated with CAR T-cells (around day 14) (Treated Group: Td) or not treated as a control (Not Treated: NT). Mice were culled when tumors reached threshold or at the end of the experiment. **A.** The tumor growth curves for the individual tumors**. B.** The mean and individual volumes at day 49**. C** The survival curves for treatment and control mice. No significant difference was seen between treatment and control mice.

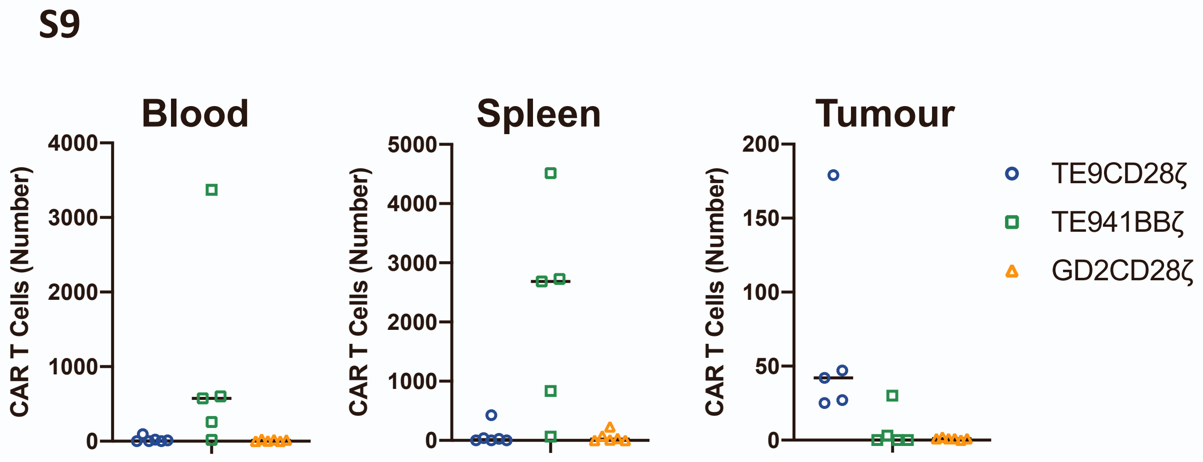

Figure S9. *TE9-28ζ CAR T Cells showed greater penetrance and survival within the tumor compared with the other treatment groups*. Blood, spleen, and tumor samples were harvested at the time of sacrifice for humane endpoints and analyzed for persistence of CAR T cells using flow cytometry. The graphs show the total number of CAR T cells detected in each sample as determined by human CD45, human CD3 and CD34 co-expression. GD2-CD28**ζ**, n = 6 for all samples, TE9-CD28**ζ** n = 6 (blood and spleen), n = 5 (tumour). TE9-41BB**ζ** n = 5 for all samples. CAR-T number is not normalized for size or volume of tissue.
