# Supplementary material for: A novel anti-B7-H3 chimeric antigen receptor from a single-chain antibody library for immunotherapy of solid cancers
Source: Mol Ther Oncolytics. 2022 Aug 25;26:429–43. doi: 10.1016/j.omto.2022.08.008 (PMC9467911; doi:10.1016/j.omto.2022.08.008)
Supplement: Document S2. Article plus supplemental information [file mmc2.pdf]

Molecular Therapy
Oncolytics
Original Article

A novel anti-B7-H3 chimeric antigen receptor from a single-chain antibody library for immunotherapy of solid cancers

Kathleen Birley,[1] Clara Leboreiro-Babe,[1] Enrique Miranda Rota,[2] Magdalena Buschhaus,[2] Artemis Gavriil,[1] Alice Vitali,[1] Maria Alonso-Ferrero,[1] Lee Hopwood,[1] Lara Parienti,[1] Gabrielle Ferry,[1] Barry Flutter,[1] Nourredine Himoudi,[1] Kerry Chester,[2] and John Anderson[1]

[1]Zayed Centre for Research, University College London, 20c Guildford Street, London WC1N 1DZ, UK; [2]The Cancer Institute, University College London, 72 Huntley Street, London WC1E 6DD, UK

B7-H3 (CD276) has emerged as a target for cancer immunotherapy by virtue of consistent expression in many malignancies, relative absence from healthy tissues, and an emerging role as a driver of tumor immune inhibition. Recent studies have reported B7-H3 to be a suitable target for chimeric antigen receptor-modified T cell (CAR-T) therapy using CARs constructed from established anti-B7-H3 antibodies converted into single-chain Fv format (scFv). We constructed and screened binders in an scFv library to generate a new anti-B7-H3 CAR-T with favorable properties. This allowed access to numerous specificities ready formatted for CAR evaluation. Selected anti-human B7-H3 scFvs were readily cloned into CAR-T and evaluated for anti-tumor reactivity in cytotoxicity, cytokine, and proliferation assays. Two binders with divergent complementarity determining regions were found to show optimal antigen-specific cytotoxicity and cytokine secretion. One binder in second-generation CD28-CD3ζ CAR format induced sustained *in vitro* proliferation on repeat antigen challenge. The lead candidate CAR-T also demonstrated *in vivo* activity in a resistant neuroblastoma model. An empirical approach to B7-H3 CAR-T discovery through screening of novel scFv sequences in CAR-T format has led to the identification of a new construct with sustained proliferative capacity warranting further evaluation.

INTRODUCTION

Immunotherapy in the form of chimeric antigen receptor (CAR) T cell technology has yielded complete clinical responses and long-term cures in many patients with otherwise refractory B cell malignancies.[1–3] Despite this progress, similar successes have not been replicated in solid tumors for several reasons, including relative absence of suitable antigen targets, and challenges of penetrance and persistence in a solid tumor environment.[4] Pediatric solid tumors create additional challenges due to a sparsity of neoantigens and their immunologically "cold," hostile microenvironments.[5,6]

B7-H3 (CD276) is a target for CAR T cell therapy in both solid and liquid malignancies arising in adults and children.[7,8] A member of the immunoglobulin superfamily and the B7 family closely related to PD-L1, B7-H3 is found on most pediatric solid cancers, with a propensity for increased expression on high-grade tumors but relatively absent from healthy cells.[7,9–14] The most common isoform of B7-H3 in humans is isoform 1 or 4Ig-B7-H3; however, alternate splicing can result in the production of 2Ig-B7-H3 (isoform 2).[9,15,16] B7-H3 evolved in its 4Ig form due to exon duplication, and its subunits V1-C1 and V2-C2 are almost identical.[17]

When originally identified, B7-H3 was thought to be involved in T cell activation, but over time the body of evidence points to its predominant role as an inhibitor of the innate and adaptive immune system.[9,18,19] The mechanism through which B7-H3 acts is poorly understood and, although some receptors have been implicated, no study has conclusively identified the receptor or receptors through which B7-H3 signals.[20,21] In addition, B7-H3 is thought to have non-immunological roles in cancer progression and high expression is associated with increased invasion, metastasis, resistance to chemotherapy, and a poorer prognosis.[22–25]

Other anti-B7-H3 CAR T cell products have been reported and translated into clinical trials. These predominantly incorporate a single-chain Fv fragment (scFv) adapted from a monoclonal antibody, such as MGA271 and 376.96.[26,27] Preclinical studies of anti B7-H3 CAR-T using these antibody-adapted scFvs show their cytotoxic capacity against a range of solid tumors *in vitro* and in animal models.[7,12,14]

Neuroblastoma is the most common extracranial solid tumor of childhood. Although improvements have been seen in treatment,

Received 14 April 2022; accepted 19 August 2022;
https://doi.org/10.1016/j.omto.2022.08.008.

Correspondence: John Anderson, Zayed Centre for Research, University College London, 20c Guildford Street, London WC1N 1DZ, UK.
E-mail: j.anderson@ucl.ac.uk
Correspondence: Kerry Chester, The Cancer Institute, University College London, 72 Huntley Street, London WC1E 6DD, UK.
E-mail: k.chester@ucl.ac.uk

This is an open access article under the CC BY license (http://creativecommons.org/licenses/by/4.0/).

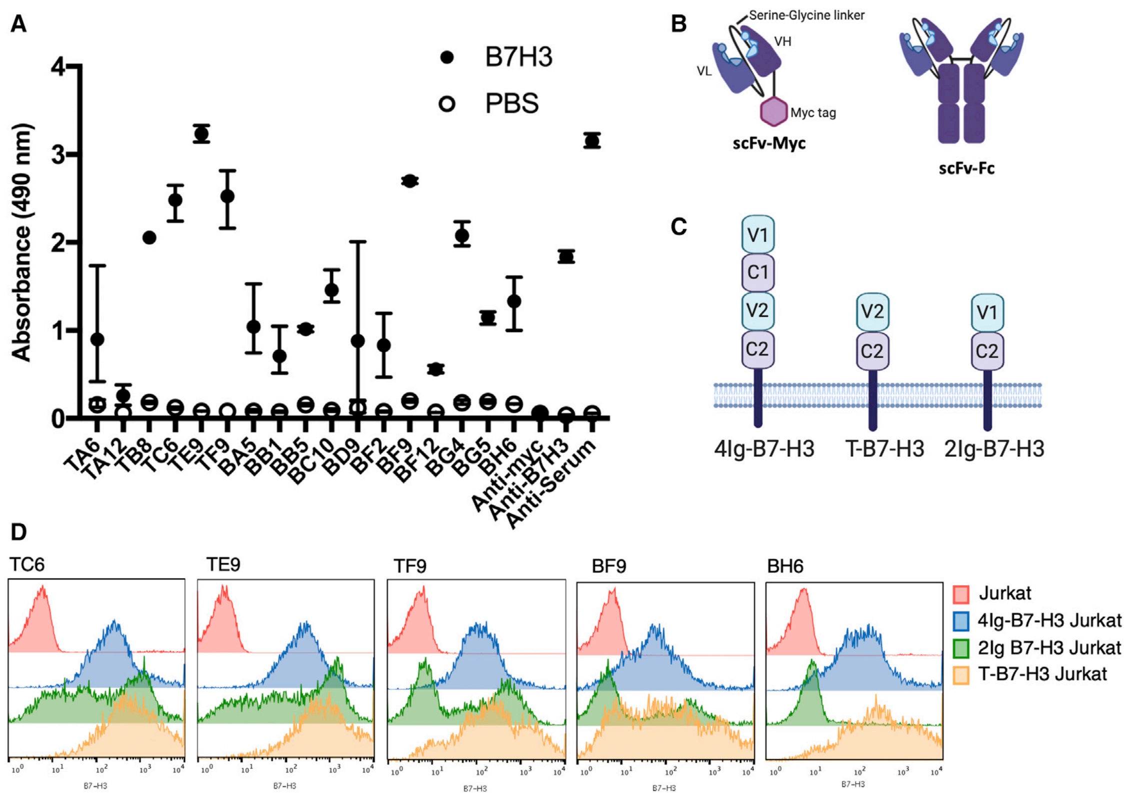

Figure 1. Anti-B7-H3 scFvs were identified that showed binding against plate bound and cell bound B7-H3

(A) Bacterial clones demonstrating an anti-B7-H3 response in screening were regrown and retested in triplicate. Following induction of scFv-myc production, bacterial supernatant was tested in an ELISA against recombinant B7-H3 or PBS as a negative control. A commercial anti-B7-H3 and the serum from the immunized mouse were used as positive controls and a secondary only (anti-myc) as a negative control (mean and SD, n = 3). (B) The structure of the scFv-myc and scFv-Fc proteins. (C) Jurkat cells were transduced with one of three isoforms of B7-H3: human 4IgB7-H3, human 2IgB7-H3, and artificial T-B7-H3. (D) The binding of scFv-Fc against different cell bound isoforms of B7-H3. Representative 1 of 2.

children with high-risk disease continue to have a poor prognosis with a high rate of relapse and significant treatment-associated morbidity.[28] The 5-year survival in this group remains less than 50% despite aggressive multimodal therapy.[29] Several phase I trials of anti-GD2 CAR-T cell therapy in neuroblastoma patients have collectively showed some clinical responses but these were short lived. Importantly, the therapy was well tolerated, and no severe toxicity was noted, identifying CAR-T as a promising approach for neuroblastoma but requiring further refinement.[30]

Since scFvs used in CARs are typically adapted from existing monoclonal antibodies and the binding kinetics and spatial aspects of antigen binding sites governing a successful CAR are poorly understood, we set out to screen libraries of scFvs to identify an optimal binder for anti-B7-H3 CAR-T. From a panel of 17 anti-B7-H3 scFvs, we selected a lead binder, TE9. Using *in vitro* assays, we identified a second-generation CAR structure TE9-CD8 hinge-transmembrane (H/Tm)-CD28-CD3ζ as having optimal anti-tumor effects against neuroblastoma cell lines. In repeat challenge and animal studies this CAR showed tumor retardation and penetrance superior to an anti-GD2-CAR recently used in clinical trial in neuroblastoma.

RESULTS

Development of novel anti-B7-H3 antibodies in single-chain format

The targeting of B7-H3 cancer antigen by T cells engineered to express CARs has shown great promise in preclinical models and is being translated into clinical studies. Thus far, most studies have used repurposed antibodies in which scFvs have been derived from existing monoclonal antibodies. To generate novel B7-H3 binders potentially more finely tuned for CAR-T applications, our strategy was to screen scFvs derived from a phage library by panning with human B7-H3, and then cloned directly into CAR-T format for empirical comparison of CAR-T effector function (Figure S1). The size of the immunized library and the panned libraries were estimated by serial dilution (Figure S1D).

Seventeen binders were identified by ELISA screen (Figure 1A) and sequence analysis indicated a high degree of diversity (Figure S2). Ten scFvs were selected for production in scFv-Fc format based on binding to plate bound B7-H3 in ELISA and genetic heterogeneity of the clones. Of 10 scFv-Fc fusion proteins, 5 (TE9, TC6, BH6, TF9, and BF9) were selected for further evaluation in CAR-T format

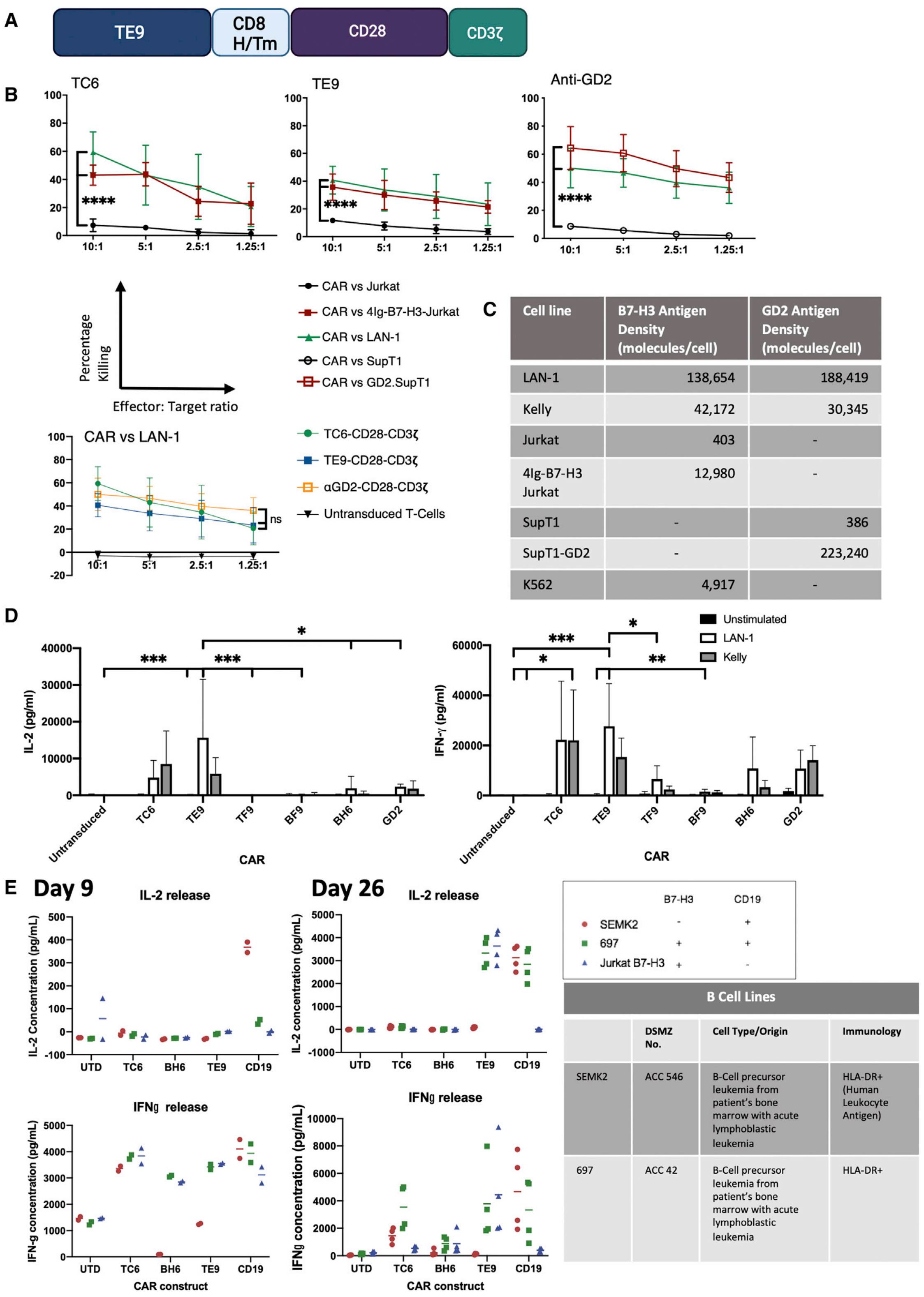

Cell line	B7-H3 Antigen Density (molecules/cell)	GD2 Antigen Density (molecules/cell)
LAN-1	138,654	188,419
Kelly	42,172	30,345
Jurkat	403	-
4Ig-B7-H3 Jurkat	12,980	-
SupT1	-	386
SupT1-GD2	-	223,240
K562	4,917	-

	B7-H3	CD19
SEMK2	-	+
697	+	+
Jurkat B7-H3	+	-

B Cell Lines

	DSMZ No.	Cell Type/Origin	Immunology
SEMK2	ACC 546	B-Cell precursor leukemia from patient's bone marrow with acute lymphoblastic leukemia	HLA-DR+ (Human Leukocyte Antigen)
697	ACC 42	B-Cell precursor leukemia from patient's bone marrow with acute lymphoblastic leukemia	HLA-DR+

(legend on next page)

based on the pattern of strength and specificity of binding to B7-H3 isoforms (isoform 1, isoform 2, or the artificially truncated isoform T-B7-H3 that had been used as the immunogen) by flow cytometry (Figures 1B–1D). Binding to the 4Ig version (isoform 1) was particularly important in selection of binders since it is the predominant human isoform. Five other scFvs showed less cell-bound B7-H3-specific binding and were not taken forward (Figure S3A). Three of the anti-B7-H3 binders (TE9, TC6, and TF9) bound to both human isoforms of B7-H3. BF9 showed equivocal binding to 2Ig-B7-H3 and BH6 showed specificity for 4Ig-B7-H3 (Figure 1D). The binders, TE9, TC6, and BH6 were produced in whole-antibody format. These antibodies showed specific binding against B7-H3 but not against other members of the human B7 family in ELISA (Figure S4B). BH6 bound both human and mouse B7-H3, but TE9 and TC6 were specific for human and cynomolgus monkey (Figure S4). TE9 and BH6 in whole-antibody format showed similar antigenic specificities to neuroblastoma and synthetic cell lines as commercial anti-B7-H3 monoclonal antibodies. Whole-antibody BH6 showed specificity for 4Ig-B7-H3 (Figure S5).

B7-H3 binders in CAR-T format display a range of antigen-specific effector function

The five candidate scFv sequences were evaluated for ability to confer antigen-specific T cell function in second-generation CD8H/Tm-CD28-CD3ζ (28ζ) CAR format comprising the CD8α H/Tm sequences and CD28 and CD3ζ signaling domains (Figure 2A). CARs were evaluated for effector function by culturing with neuroblastoma cells naturally expressing B7-H3 and assessing cytotoxicity and cytokine secretion (Figures 2B–2D). All five CAR-T constructs showed adequate transduction efficiency in human T cells (Figure S3B). Two binders (TE9, TC6) showed significant cytotoxicity specific for B7-H3-expressing target cells in 4-h killing assays (Figure 2B) and they also showed the greatest degree of cytokine response to neuroblastoma targets. CARs expressing the anti-B7-H3 binders TF9, BF9, and BH6 did not demonstrate significant cytotoxicity or cytokine production in short-term co-cultures (Figures S3C and 2D).

To determine how CARs behaved during longer-term co-cultures, they were evaluated as CAR-T in a repeat antigenic challenge assay during which CAR-T cells received four stimulations of irradiated tumor cells over 4 weeks. Three of our anti-B7-H3 binders were compared with the FMC63 anti-CD19 CAR-T constructs. These experiments showed that TE9-28ζ and CD19-28ζ CAR-T cells had sustained capacity to produce IL-2 in response to a fourth rechallenge with B7-H3-positive leukemia cells, while TC6 and BH6 CAR-T cells had become non-responsive by the fourth stimulation (Figure 2E). We noted that the IL-2 response at day 9 was only modest against these leukemia targets, but given strong responses against neuroblastoma and strong IL-2 response by fourth rechallenge, the TE9 binder was therefore selected for further optimization of CAR-T function.

CD28 costimulation and CD8 H/Tm impart sustained cytokine production to TE9 CAR-T cells

We next compared CD28 and 4-1BB endodomains combined with a CD8 H/Tm (Figure 3A) by assaying effector function against B7-H3-expressing neuroblastoma cells. Transduction efficiency between 20% and 80% was observed with the two constructs (Figure 3B). Following an 18-h co-culture of CAR-T cells with neuroblastoma target cells, cytotoxic degranulation as determined by CD107a as well as upregulation of CD25 and CD69 activation markers following addition of targets, was non-significantly higher in CD28ζ than 4-1BBζ constructs (Figure 3C). CD28ζ CAR-T cells did display significantly higher levels of CD69 and CD107a compared with untransduced T cells, which 4-1BBζ constructs did not (Figure 3C). In short-term co-cultures, TE9-28ζ generated more interferon-γ (IFN-γ) and significantly greater IL-2 than its 4-1BB counterpart (Figure 3D). 4-1BB endodomains in CAR-T cells have been well described to confer ability for longer-term effector function on antigen rechallenge.[31] We therefore assessed cytokine production following rechallenge with neuroblastoma cells 7 days after initial antigenic challenge. Here, the TE9-BBζ CARs induced lower levels of both IL-2 and IFN-γ (Figure 3E). The greater activation with the CD28 endodomain was reflected in greater upregulation of activation/exhaustion markers (Figure S6).

Previous studies have indicated that a CD28 H/Tm confers greater sensitivity to target antigens than CD8 H/Tm.[8,32] We therefore compared TE9-28ζ with these two H/Tm arrangements, both of which were expressed on T cells at similar levels (Figures 4A and 4C). We stimulated the respective CAR-T constructs with decreasing concentrations of recombinant B7-H3 and demonstrated only a marginal enhanced IFN-γ and IL-2 response of the CD28 H/Tm construct, which was most marked for IFN-γ and at lowest antigen concentrations (Figure 4B). These proof-of-concept data encouraged us to evaluate cells of known and more physiologically relevant ranges of B7H3 expression. We therefore next evaluated cytokine production after 18 h co-culture with neuroblastoma cells LAN-1 and Kelly which have different levels of B7-H3 expression, and K562 cells which have a low expression of B7-H3 (Figure 2C). In addition, we evaluated cytokine production and proliferation after 7 days incubation followed by an antigen rechallenge, with cytokine analysis 24 h later. No statistically significant difference was noted between the CARs, both of which demonstrated

Figure 2. The anti-B7-H3 CAR-T cell shows T cell effector functions similar to anti-GD2 and anti-CD19 CAR-T cells

(A) The second-generation CAR design used in this study incorporating the CD8 H/Tm and the CD28-CD3ζ endodomains. (B) Cr^{51} cytotoxicity assay of lead CAR-T cells against isogenic B7-H3 ± cell lines and LAN-1 cells. Comparison made with anti-GD2 CAR-T cells against isogenic GD2 ± cell lines and LAN-1 (mean and SD, n = 3; ns $p \geq 0.05$, ****$p < 0.0001$). (C) B7-H3 and GD2 antigen density of cell lines used in the study measured using a Quantbrite antigen quantification kit (BD Bioscience). (D) CAR-T cells were cultured with LAN-1, Kelly, or no antigen stimulus for 18 h. IL-2 and IFN-γ were measured in supernatant by ELISA (mean and SD, n = 3–5; *$p \leq 0.05$, **$p \leq 0.01$, ***$p \leq 0.001$). (E) Antigen-specific cytokine response at the end of the tumor rechallenge assay at day 9 (24 h after second stimulation) and day 26 (24 h after the fourth stimulation). Mean and range of data are from two donors. Each sample analyzed once in duplicate.

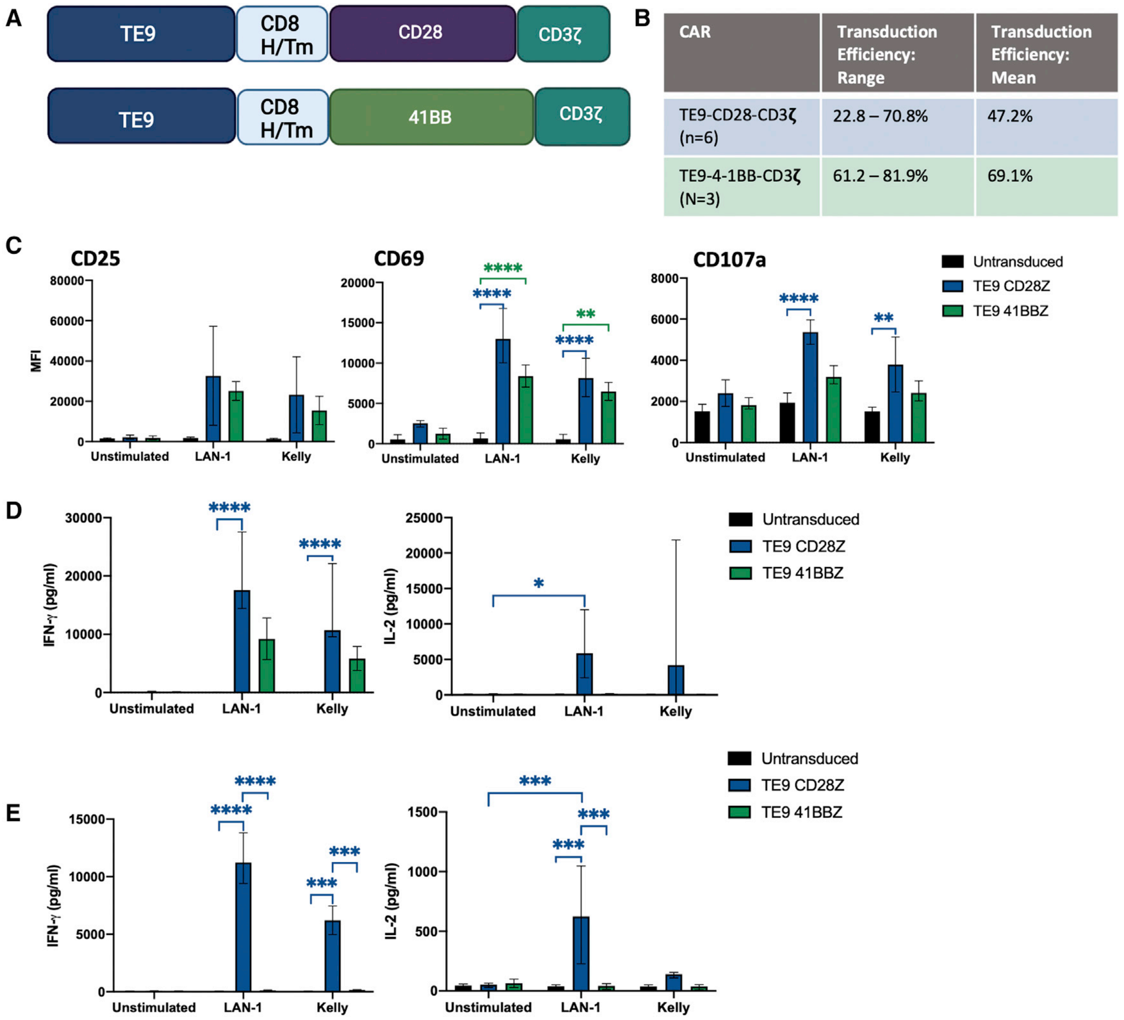

Figure 3. TE9-28ζ CAR T cells show superior cytokine production compared with TE9-41BBζ CAR T cells

(A) Diagram of the second-generation CD28-CD3ζ and 4-1BB-CD3ζ CAR constructs used in this study. (B) Transduction efficiency of TE9-28ζ (mean and range, n = 6) or TE9- 41BBζ (mean and range, n = 3). (C) CAR-T cells or untransduced cells were cultured with cells containing antigen targets (LAN-1 or Kelly) or no antigen targets (unstimulated) for 18 h. Cells were stained for CD107a, CD69, and CD25 (mean and range, n = 3; *p ≤ 0.05, **p ≤ 0.01, ****p < 0.0001). (D) CAR-T cells or untransduced cells were cultured with LAN-1 or Kelly target cells or no antigen stimulus for 18 h. Cells were pelleted and supernatant used in ELISA compared with standard values of IL-2 or IFN-γ (mean and range, TE9-28ζ, untransduced, n = 6. TE9-BBζ, n = 3; *p ≤ 0.05, ****p < 0.0001). (E) CARs and untransduced T cells were cultured with LAN-1 or Kelly targets, or US for 7 days then re-stimulated with fresh antigen targets and incubated for a further 24 h. IFN-γ and IL-2 production were measured using ELISA (mean and range, n = 3; ***p ≤ 0.001, ****p < 0.0001).

antigen-specific effector function, although the CD28 H/Tm led to higher mean IFN-γ response in the presence of the B7-H3-low K562 cells (Figures 4D–4F). Similarly in short-term killing against LAN-1, there was non-significant increased cytotoxicity with the CD8 H/TM (Figure 4G). Hence, in contrast to work with other binders, in our hands, the CD28 H/Tm only provided marginal increased sensitivity to low antigen target. Healthy cells may express low levels of B7-H3 resulting in on target:off tumor toxicity. Given the minimal increase in function observed using the CD28H/Tm, the decision was made continue with the CD8H/Tm this construct was felt to give the best balance of efficacy and safety.

TE9 shows comparable levels of cytokine secretion, cytotoxicity, and proliferation to other anti-B7-H3 scFvs

TE9 was benchmarked against two other anti-B7-H3 scFvs used in the literature in CAR format; MGA271 and 376.96.[7,12,14] These scFvs were cloned into the SFG CAR backbone with a CD8H/Tm-CD28-CD3ζ format for exact side-by-side comparison with TE9-28ζ (Figure 5A). Cytotoxicity was assessed against LAN-1 cells in a chromium release assay and no significant difference was seen between the three scFvs (Figure 5B). CAR-T cells and untransduced cells were cultured with neuroblastoma cell lines or no antigen stimulus for 18 h. Cell supernatant was collected for analysis of IL-2 and IFN-γ production

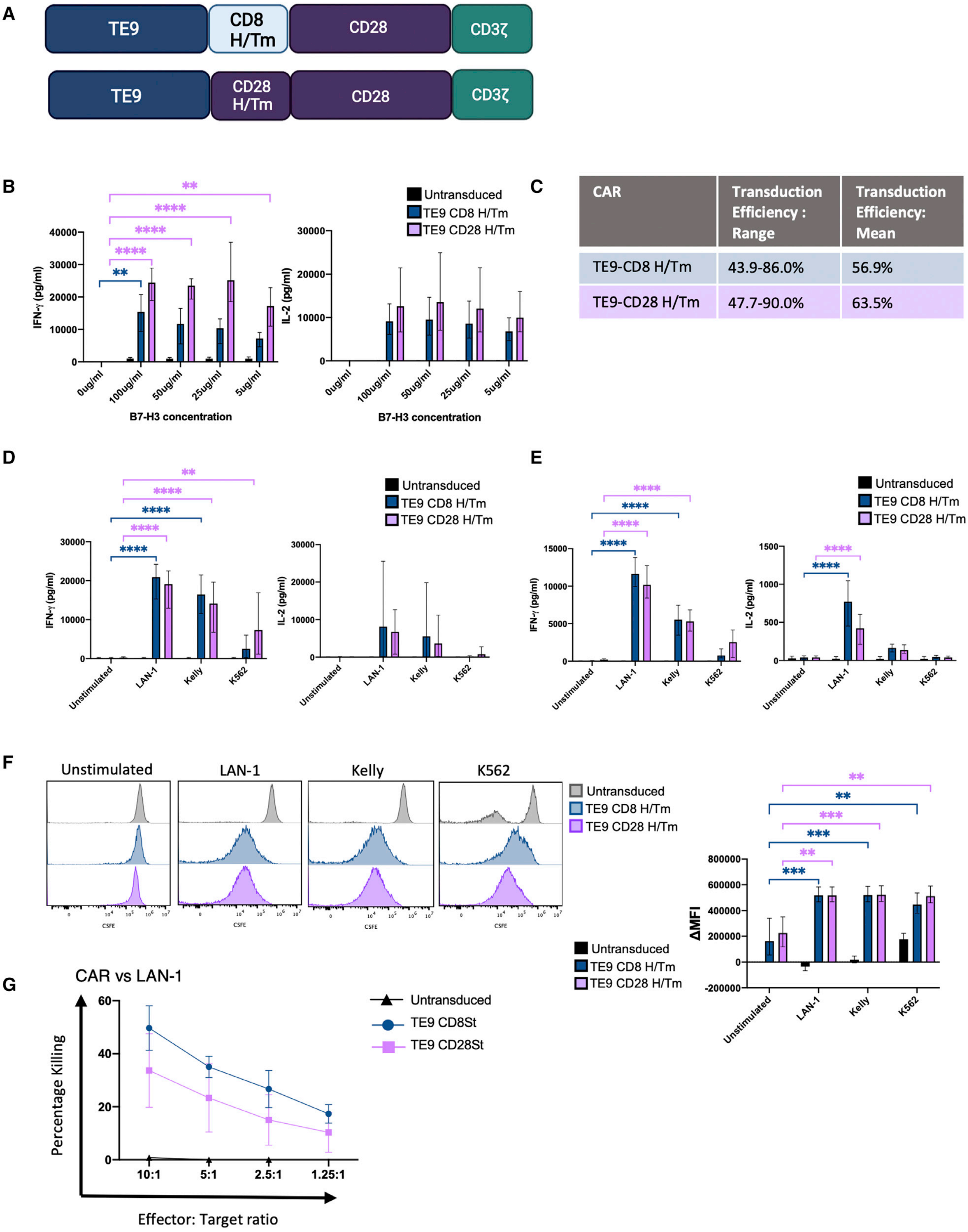

(legend on next page)

with ELISA, and cells were stained for T cell activation markers CD25 and CD69 and the degranulation marker CD107a (Figures 5C and 5D). All three CAR show similar levels of cytokine production and activation. Although some significant differences were noted between the CAR-T cells and the untransduced cells, no significant differences were noted between the CAR-T constructs themselves.

CAR-T cells and untransduced cells were stained with CSFE and cultured with neuroblastoma targets for 7 days and then re-stimulated with a fresh antigen-positive target cells. Proliferation was measured using CSFE dilution on the flow cytometry and cell supernatant was collected for evaluation cytokine production with ELISA (Figures 5E and 5F). Again, CAR-T cells showed similar levels of proliferation and cytokine production with some significant differences compared with untransduced T cells, but no differences were noted between the CARs themselves. It is not possible to extrapolate from this data how TE9 would compare with the optimized MGA271 and 376.96 CAR-T cells as changes in the backbone and key components will affect CAR function. MGA271 CAR-T cells have predominantly been tested with 41BB-CD3ζ endodomains, for example, Theruvath and co-workers.[12,14] However, these data suggest that, in our hands, TE9-28ζ has a similar level of function to other anti-B7-H3 CAR-T cells in short-term *in vitro* assays.

TE9-28ζ with CD8 H/Tm shows enhanced long-term proliferation in *in vitro* testing and effector function in an *in vivo* neuroblastoma model

We confirmed that TE9 with CD8 H/Tm was effective in long-term stimulation in stress conditions by challenging at weekly intervals with irradiated neuroblastoma cells and comparing with CAR-T cell survival of an anti-GD2 second-generation CAR that has been shown to have clinical function but limited *in vivo* persistence.[30] Previous work has demonstrated that incorporation of IL-2 receptor β chain into second-generation anti-CD19 CAR endodomains, combined with mutation of CD3ζ residues to enhance STAT3 phosphorylation, leads to longer-term persistence.[33] We evaluated whether these modifications could enhance persistence to the TE9 anti-B7-H3 CAR-T cells in response to neuroblastoma challenge (Figure 6). We assessed response to both Kelly and LAN-1 neuroblastoma targets that express similar antigen levels of both GD2 and B7-H3 (Figure 2B). While the anti-GD2 CAR-T cells failed to expand or to produce cytokines after the second stimulation, the TE9-28ζ CAR-T persisted for 4 weeks and continued to generate both IL-2 and IFN-γ at levels significantly above background (Figure 6). In our hands, the addition of the cytokine signaling domains was consistently inferior to conventional second-generation TE9-28ζ cells for both persistence and cytokine production (Figures 6 and S7).

Although Kelly cells have a lower level of antigen expression than LAN-1 cells (Figure 2C), TE9-CD28ζ CAR-T cells and TE9-IL2R CAR-T cells produced similar levels of cytokines when cultured with either cell line and show greater proliferation in culture with Kelly. This could be because LAN-1 cells are more inhibitory than Kelly cells, suppressing T cell effector function, or it could be because cells cultured with a moderate level of target antigen are less prone to exhaustion. Further testing looking at T cell dysfunction in the form of exhaustion markers and metabolic function could be used to investigate this further.

To determine if the long-term persistence of TE9-28ζ during repeat stimulation in stress conditions translated into effective *in vivo* function we used the LAN-1 subcutaneous neuroblastoma model, which we demonstrated previously to have been resistant to growth inhibition by GD2-28ζ CAR-T cells using the same GD2 CAR construct that we have recently demonstrated to lead to short-term clinical activity in patients (Figure S8).[30] To provide further comparative data on CD28 versus 4-1BB endodomains in the context of longer-term CAR-T function than had been evaluated *in vitro*, the two endodomains were compared side by side. Mice treated with TE9-28ζ CAR-T cells showed enhanced survival and shrinkage of small established tumors compared with TE9-BBζ- and anti-GD2 CAR-T-treated mice (Figure 7). We did not observe reduction in B7-H3 or GD2 antigen expression in treated mice, and hence have not found evidence that antigen loss could account for treatment failure, albeit in the context of a single model evaluated (Figure 7F). Blood, spleen, and tumor samples were harvested when tumors reached a threshold size or at the end of the experiment. Samples from all mice were processed; however, one mouse treated with TE9-28ζ had no detectable tumor at the end of the experiment and samples from one mouse treated with TE9-BBζ had to be discarded due to a processing error. Samples with <100 human CD45 cells were not included as numbers were felt to be too low to be accurate. Human CD45 cells were then further evaluated for CAR+ cells through staining for the detection marker CD34. Data are presented as total number of CAR-T cells in each respective organ. Higher total numbers of CAR+ cells were seen in the blood and spleen of mice treated with TE9-BBζ while

Figure 4. Second-generation CAR T cells with a CD28 H/Tm show superior cytokine production and proliferation in the presence of low antigen expression
(A) A schematic of the second-generation TE9-CAR T cells with the CD8 and CD28 H/Tm. (B) Cytokine production by T cells transduced with second-generation CARs containing either a CD8 H/Tm or a CD28 H/Tm or untransduced. T cells were incubated overnight with different concentrations of plate bound B7-H3 and the supernatant analyzed for cytokine production (mean with range, n = 3; $^{**}p \leq 0.01$, $^{****}p < 0.0001$). (C) The transduction efficiency of TE9-CD8 St and TE9 CD 28 H/Tm (mean with range, n = 6). (D) TE9 CAR T cells with either a CD28 H/Tm or CD8 H/Tm were incubated with LAN-1, Kelly, K562, or no antigen stimulus for 18 h. Supernatant was used to quantify cytokine production using ELISA (mean and range, n = 6; $^{**}p \leq 0.01$, $^{****}p < 0.0001$). (E) After 7 days of co-culture, T cells were re-stimulated with fresh target cells or no antigen targets. Supernatant from 7-day co-cultures was used to quantify cytokine production using ELISA (mean and range, n = 4; $^{****}p < 0.0001$). (F) TE9 CAR T cells with either a CD28 H/Tm or CD8 H/Tm were stained with CSFE and incubated with LAN-1, Kelly, K562, or no antigen stimulus for 7 days. Histograms show dilution of CSFE due to proliferation against different targets (representative 1 of 3). The ΔMFI (median fluorescence intensity) was calculated as the difference between the MFI of the test condition compared with the unstimulated untransduced control (mean and range, n = 3; $^{**}p \leq 0.01$, $^{***}p \leq 0.001$). (G) CAR T cells with the CD8 and CD28 H/Tm domains and untransduced T cells were evaluated for cytotoxicity against LAN-1 target cells in a chromium release assay (mean and SD, n = 3).

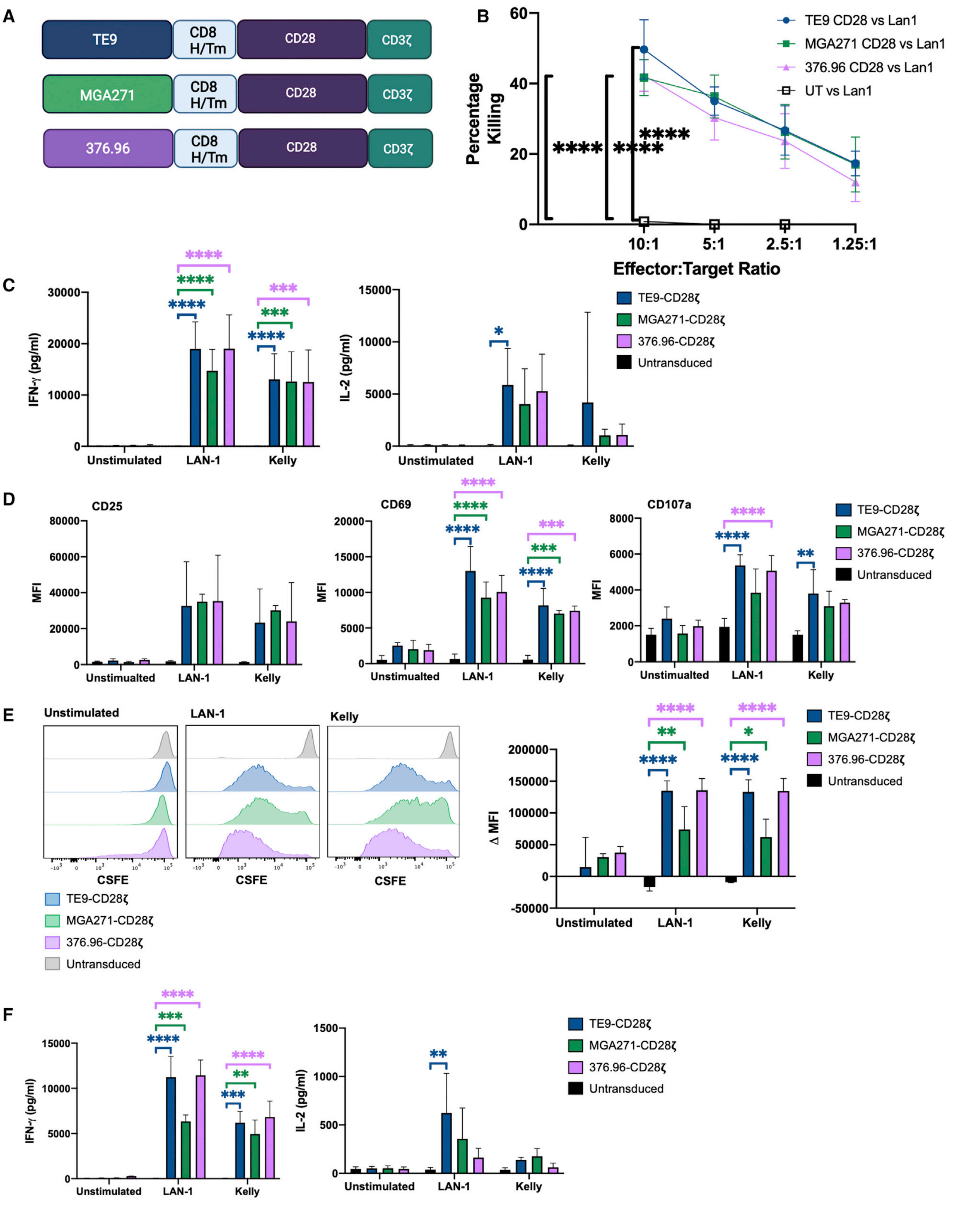

(legend on next page)

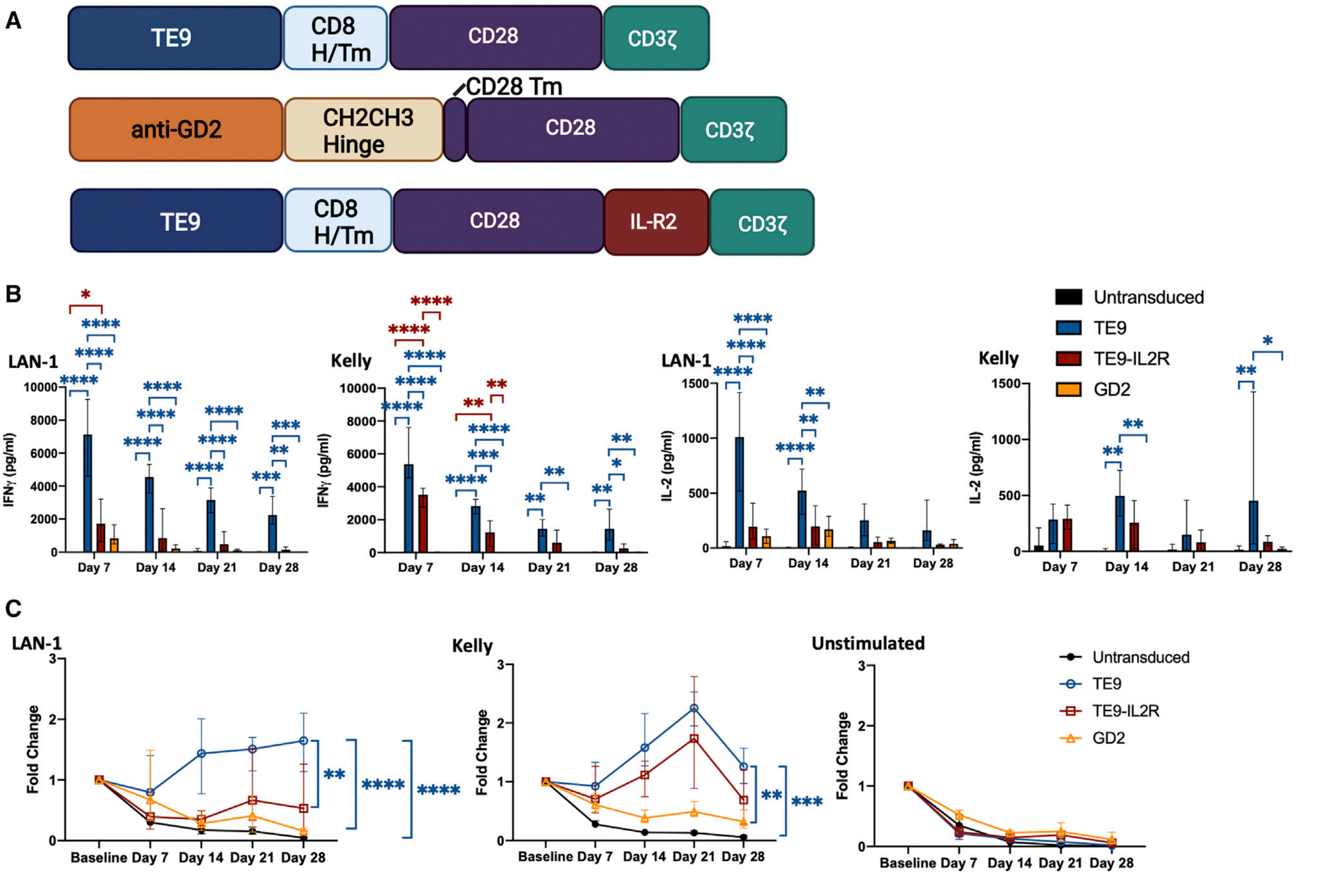

Figure 6. TE9-28ζ shows superior expansion and cytokine production in long-term assays compared with GD2-28ζ
CAR T cells were transduced with TE9-28ζ (TE9), TE9-28-ILR2ζ (*TE9-ILR2*), or *GD2-28*ζ (*GD2*). (A) A schematic of the CAR T cells used in this study. (B) CAR T cells or untransduced cells were cultured with either LAN-1, Kelly, or no antigen stimulus. Each week, cells were given a fresh antigen stimulus, cultured for a further 24 h then analyzed. The production of IFN-γ and IL-2 was determined using ELISA after each antigen stimulus (mean and range, n = 4; *p ≤ 0.05, **p ≤ 0.01, ***p ≤ 0.001, ****p < 0.0001). (C) The proliferation as measured by the fold change of CD3+ cells measured using flow cytometry. The significance is shown between cell numbers on day 28 (mean with range, n = 4; **p ≤ 0.01, ***p ≤ 0.001, ****p < 0.0001).

only 1/5 of tumors of TE9-BBζ-treated mice had detectable CAR-T cells. In contrast, detectable numbers of CAR-T cells were seen in 4/5 of tumors in mice treated with TE9-28ζ (Figure S9). This may suggest better penetrance and/or survival into LAN-1 tumors in mice treated with this CAR, although more work is needed to investigate this further. Taken together, the data indicate the ability of TE9-28ζ CAR-T cells to effect tumor shrinkage of an established and treatment refractory neuroblastoma model without evidence of the emergence of antigen loss variants.

DISCUSSION

We successfully identified 17 unique scFvs following panning of libraries derived from splenic RNA from immunized mice. The libraries following immunotube panning and bead panning were of a

Figure 5. TE9 is comparable with two previously described anti-B7-H3 scFvs in *in vitro* models
(A) CD28-CD3ζ CAR T cells were produced with the anti-B7-H3 scFvs MGA271 and 376.96. (B) Cytotoxicity of anti-B7-H3 CAR T cells or untransduced T cells measured using a chromium release assay against LAN-1 target cells (mean with SD, n = 3; ****p < 0.0001). (C) CAR T cells or untransduced T cells were cultured with LAN-1, Kelly, or no target cells (unstimulated) for 18 h. ELISA of cell supernatant was used to measure IFN-γ and IL-2 production (mean with SD, n = 6; *p ≤ 0.05, ***p ≤ 0.001, ****p < 0.0001). (D) T cells or untransduced T cells were cultured with LAN-1, Kelly, or no target cells (unstimulated) for 18 h. Cells were stained for T cell activation markers CD25, CD69, and the degranulation marker CD107a as a proxy for cytotoxicity. The MFI was measured using flow cytometry (mean with SD, n = 3; **p ≤ 0.01, ***p ≤ 0.001, ****p < 0.0001). (E) CAR T cells or untransduced T cells were stained with CSFE and cultured with LAN-1, Kelly, or no target cells for 7 days. At the end of 7 days, the MFI was measured using flow cytometry. The histogram is representative 1 of 3. The bar chart shows the change in MFI from untransduced, unstimulated cells, which is taken as 0 (mean with SD, n = 3; *p ≤ 0.05, ***p ≤ 0.001, ****p < 0.0001). (F) CAR T cells or untransduced T cells were cultured with LAN-1, Kelly, or no target cells for 7 days then re-stimulated with fresh antigen targets. Twenty-four hours after re-stimulation, cell supernatant was collected for quantification of IFN-γ and IL-2 production using ELISA (mean with SD, n = 3; **p ≤ 0.01, ***p ≤ 0.001, ****p < 0.0001).

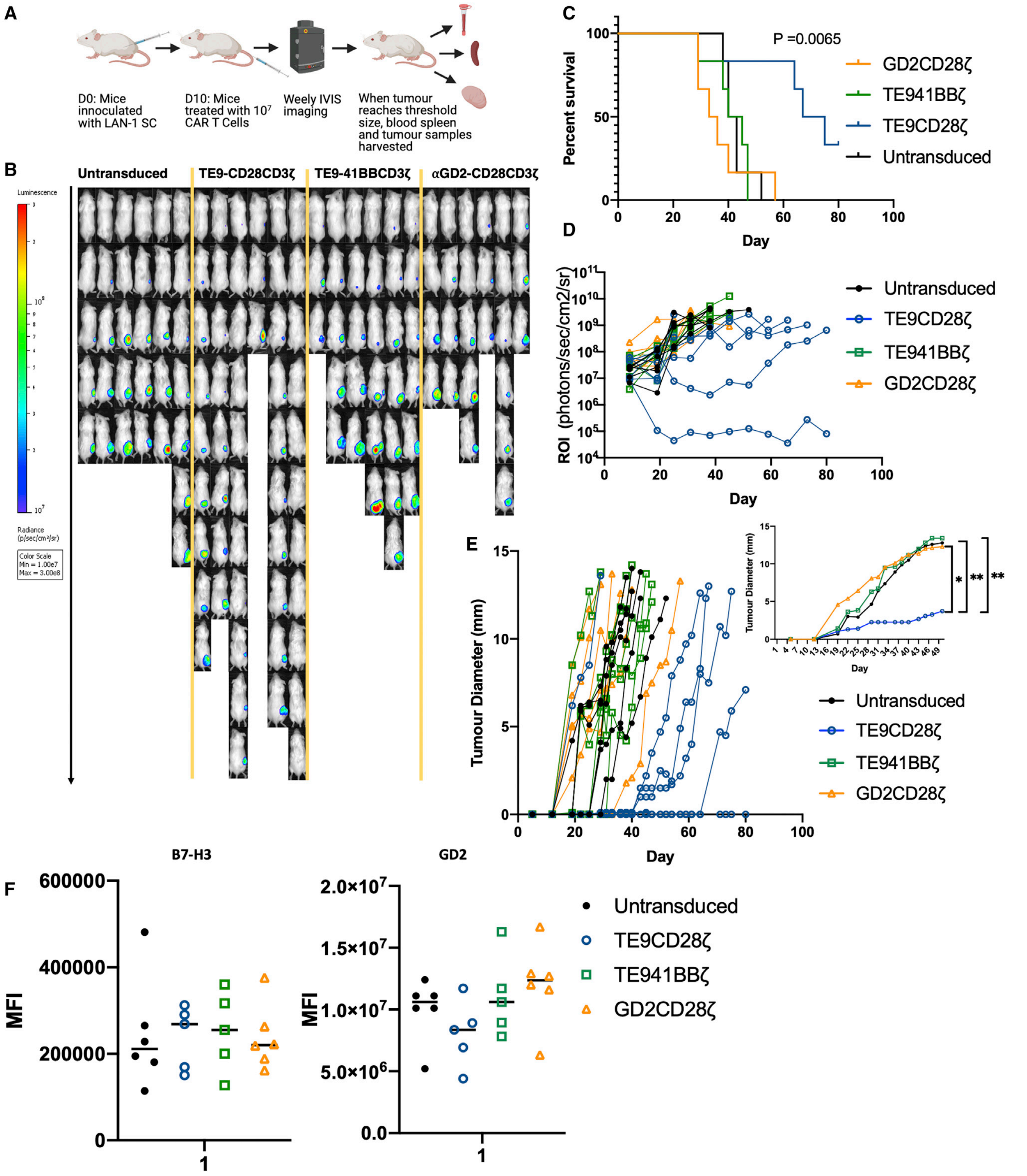

Figure 7. *In vivo* testing of TE9-28ζ, TE9-BBζ, and αGD2-28ζ

Mice treated with TE9-28ζ show increased survival and reduced tumor growth compared with other groups. (A) Experiment plan. (B) IVIS imaging. (C) Survival curve showing percentage of overall survival: analyzed and found to be significant using log-rank (Mantle-Cox) test (**$p \leq 0.01$). (D) Region of interest (ROI) measurements taken at weekly IVIS imaging (photons/second/cm^2/sr). (E) Main graph: largest tumor diameter (mm) measured with digital calipers. Insert: representation of tumor size at day 50 (mean, n = 6; *$p \leq 0.05$, **$p \leq 0.01$). (F) Antigen expression after treatment as illustrated by the MFI of fluorophores used to stain antigens in tumor samples (mean and range, mice in untransduced, and anti-GD2-28ζ groups, n = 6. Mice in TE9-28ζ group n = 5).

similar, relatively small size (Figure S1D). Although optimal diversity of a library for the generation of binders is not known, in theory, a greater diversity allows a greater range of epitope targets to be identified.[34,35] However, some sequence convergence is observed following high-throughput library screening, in part due to survival advantages conferred to the phage by the plasmid, and in part due to specific recognition of epitopes used in the screening platform. Moreover, some studies suggest that complementarity determining region-H3 diversity, usually considered important to antibody response, is not necessary to generate functional binders in phage display libraries.[36,37] In this study, the identification of a range of scFvs that could be taken forward for further testing, and at least two binders that function as CAR T cells, suggest that the panned libraries were sufficiently diverse for our purposes (Figures 1 and S2).

Ten scFvs were taken forward into scFv-Fc protein format, and showed a range of B7-H3-specific binding to cell bound B7-H3 (Figures 1D and S3A). Five of these were selected for testing in CAR-T cell format. Despite specific binding to cell bound B7-H3, only two scFvs, TE9 and TC6, showed anti-tumor activity in CAR-T cell format, as assessed by cytotoxicity and cytokine production (Figures 2B, 2D, and S3C). Further work is needed to understand the mechanics of why scFvs with apparent similar binding have very different function when applied to CARs. We hypothesized that the optimal binding kinetics for antibody binders are not the same as those for CAR-T cells due to the different spatial constraints, and it is possible that the binding sites for "CAR-resistant" scFvs prevent immune synapse formation.[38,39] Similarly, CAR receptors are known to function as dimers, with dimerization largely defined by their H/Tm and transmembrane domains.[32,40] Again, the geometry of the binding site for these scFvs may obstruct dimerization and reduce function. The second-generation CARs tested in our study comprise an scFv, H/Tm, and an intracellular domain. Each of these modules can influence CAR performance.[41] It is possible that modifications to these, particularly to the H/Tm domain, may have improved the function of CARs not taken forward for further analysis. Having identified two scFvs capable of similar levels of cytotoxicity as a previously optimized CAR, we proceeded to test CAR function in other ways.

CAR T cell therapy has been tested in pediatric neuroblastoma and, although clinical trials have generally found the treatment safe, no sustained responses have been reported.[30,42] In this study, we chose to focus on the neuroblastoma cell lines LAN-1 and Kelly for two reasons. Firstly, they display different levels of antigen to each other yet very similar levels of B7-H3 and GD2, which allowed us to benchmark our CAR-T cells against the anti-GD2 CAR, which showed objective clinical response in a recent trial.[30] Secondly, neuroblastomas and their derived cell lines are known to create inhibitory environments.[43–45] We wanted to identify a model that was resistant to treatment allowing better discrimination between alternate CAR-T for clinical translation. The inhibitory effects of LAN-1 can be seen as inhibition of killing and proliferation of untransduced T cell controls compared with control conditions (Figures 2B, 4F, and 5E).

Despite showing limited clinical response, the anti-GD2 CAR did not show efficacy against the *in vivo* LAN-1 model (Figures 7, S8, and S9) in contrast with TE9-28ζ, which was effective, although sustained control was only observed in 1/6 of mice at the end of the experiment (Figure 7). Furthermore, there was no evidence of antigen loss following TE9-28ζ CAR-T cells treatment in 4/5 of mice, suggesting that T cell persistence is an early cause of CAR T cell failure in this model (Figures 7F and S9). For clinical success, CAR-T cells need to overcome the inhibitory tumor environment, and lack of CAR T cell persistence is a known reason for treatment failure.[4] We tested our CAR-T cells in two different long-term or stress assays. When comparing with the FMC63 anti-CD19 CAR-T construct, by the end of 4 weeks only TE9-28ζ the anti-CD19 CAR-T cells were capable of IL-2 production in response to antigen stimulation. Of note, hematological malignancies, especially leukemias, may display differences in their microenvironment compared with solid tumors that influence T cell persistence.[46] TE9-CD28 CAR also maintained significantly greater proliferation over 4 weeks of re-stimulation compared with the anti-GD2-CAR.[30] This was the primary reason TE9 was selected as our lead binder.

Further modifications to the CAR structure were made to enhance T cell persistence in the form of the 4-1BB costimulatory endodomain and a truncated ILR2 endodomain with a mutated CD3ζ domain (Figures 3A and 6A). As described, in our hands, the TE9-ILR2 CAR did not show superiority over TE9-28ζ and was not taken forward for further evaluation.[33] 4-1BB is associated with reduced exhaustion and enhanced persistence in second-generation CAR-T cells.[31] Against the neuroblastoma cell lines included in this study, TE9-BBζ showed some anti-tumor activity at the early time point of 18 h but minimal cytokine production at 7 days (Figure 3). Consideration was given to long-term *in vitro* assays, but the decision was made to proceed directly to *in vivo* testing instead for more definitive results. TE9-BBζ CAR-T cells showed greater persistence in the blood and spleen compared with the other CARs, but CAR-T cells were only detectable in the tumors of 1/5 of mice and no growth retardation or survival advantage was noted in the TE9-BBζ treatment group (Figures 7 and S9). It is possible that the fast-acting CD28 endodomain is necessary to overcome the inhibitory microenvironment created by LAN-1 cells. Further work, including looking at the functionality and exhaustion profile of T cells found in the tumor microenvironment, is needed to test this hypothesis.

Further modifications to TE9 included testing of the CD28 H/Tm domain, which is known to improve effectiveness against low antigen density targets.[8,40] We substituted the CD8 H/Tm to the CD28 H/Tm and showed increased sensitivity of CAR-T cells against low levels of plate-bound antigen and low-density cell lines, particularly in the context of IFN-γ secretion. Although our findings supported previous work, the extent of enhanced function with CD28 H/Tm was of modest magnitude, suggesting that other factors, such as the scFvs, also impact antigen sensitivity. The optimal CAR must strike a balance between targeting malignant cells, which often display heterogeneous levels of target antigens and avoiding healthy cells, which may

display antigens at low levels. As our CD8 H/Tm displayed good levels of activity against antigen-dim tumor cells, we elected to move forward with this structure as we deemed this the safest approach without a large compromise in efficacy.

Other anti-B7-H3 scFvs have been adapted from anti-B7-H3 antibodies and used to generate anti-B7-H3 CAR-T cells. Such scFvs include MGA271 and 376.96.[7,14] Both these CAR-T cells have shown *in vivo* activity against B7-H3-positive tumors and are currently being evaluated in clinical trials. We cloned these scFvs into our CD8H/Tm-CD28-CD3ζ and compared their function against that of TE9 in short-term *in vitro* assays (Figure 5). In such tests, all three CAR-T cells perform similarly, and little difference is noted between the CARs. When described in the literature, both MGA271 and 376.96 will have been tested in their optimal structures. For example, MGA271 is usually tested with the 4-1BB-CD3ζ endodomains.[12,14] Therefore, it is difficult to comment on relative utility of alternate scFvs without further testing, but it is of encouragement that TE9-28ζ performs at least as well in our hands.

In this study, we successfully used phage display technology to identify a range of scFvs that could be rapidly tested in CAR format. Having established a lead scFv, we could then test further modifications to the CAR structure. Given the need for new treatments, particularly for childhood solid tumors like neuroblastoma, the ability to rapidly evaluate preclinical modifications may increase the pace at which therapies can be translated into the clinical setting. Furthermore, we have identified a new anti-B7-H3 scFv that demonstrates *in vitro* persistence and *in vivo* tumor control and penetrance, warranting further evaluation.

MATERIALS AND METHODS

Cells and culture conditions

Cell lines used in this study were obtained from the following suppliers: Jurkat (ATCC), 293 T (ATCC), CHO (Thermo Fisher Scientific), MEXi 293E (IBA), 293F (Thermo Fisher Scientific), LAN-1 (ECACC), Kelly (gift from Andrew Stoker, UCL), SupT1 (ECACC), and K562 (gift from Bobby Gaspar, UCL). CHO were cultured in CHO culture medium (Gibco) + 8 mM GlutaMAX (Gibco) + 0.4 mM hypoxnthine + 0.32 mM thymidine (Gibco). MEXi 293E cells were cultured in MEXi cultivation medium (IBA) + 50 mg/L geneticin and 8 mM GlutaMAX. 293F cells were cultured in Freestyle 293 Expression medium (Thermo Fisher Scientific). All three of these cells were cultured at 37°C in 5% CO_2 in an orbital shaker. A temperature of 32°C was used for protein production. A total of 293 T cells were grown in IMDM (Sigma) with 10% FCS (Gibco) and 100 U penicillin/0.1 mg streptomycin/L. They were grown at 37°C in 5% CO_2. The remainder of the cells were grown in RPMI (Sigma) + 10% FCS 100 U penicillin/0.1 mg streptomycin/L at 37°C in 5% CO_2.

γ-Retroviral transduction

A total of 293 T cells were plated at 1.5×10^6, 24 h before transfection. Cells were transduced using GeneJuice (Merck) with gene of interest expression cassette and helper plasmids env (RD114), gagpol (PegPam-env). Supernatant-containing retrovirus was harvested at 48 and 72 h. For stable transduction, target cells were plated on 24-well plates coated in retronectin (Takara) and incubated with retroviral supernatant for 72 h.

Production of the B7-H3 phage display library

Jurkat cells were stably transduced using γ-retroviral transduction to produce recombinant B7-H3-mouseFc protein. Protein was produced in a bioreactor and purified on protein A columns. Three BALB/cJ mice were injected with recombinant protein. Serial serum extractions confirmed seroconversion by flow cytometry. Splenic mRNA was extracted using RNeasy Mini Kit (QIAGEN). mRNA was reverse transcribed (Superscript III Reverse Transcriptase, Invitrogen) and then amplified via PCR (Amplitaq Polymerase, Applied Biosystems). Further PCR reactions were used to connect heavy- and light-chain DNA with a serine glycine linker. Amplified DNA was first cloned into an intermediary pSP73 vector before being cloned into the pHEN vector. *E. coli* were transduced using electroporation.

Panning of the B7-H3 library

4Ig-B7-H3 cDNA was purchased (Sinobiological) and cloned into two vectors to produce B7-H3-Histag and B7-H3-Streptag. CHO cells and MEXi293E cells, respectively, were transiently transduced with these constructs. Cells were cultured until there was a drop in their viability and protein filtered from cell supernatant using HiTrap MabSelect Protein-A Columns (Cytiva) or Strep-Tactin XT:Twin Strep-tag purification columns (IBA).

2TY medium was inoculated with *E. coli*. Bacteria were cultured until the OD was 0.5. Bacteria were infected with the M13KO7 Helper Phage (New England Biolabs) and incubated overnight. Bacteria were removed by centrifugation and phage particles in the supernatant precipitated with PEG 600/2.5 M NaCl. After washing, the phage were re-suspended in sterile water.

Immunotubes were incubated with B7-H3-Histag and MagStrep "type 3" XT beads (IBA) were incubated with B7-H3-Streptag overnight at 4°C to coat tubes/beads. Tubes/beads were washed ×3 in PBS and blocked by incubating with Marvel Milk for 2 h at room temperature. Precipitated phage was blocked with Marvel Milk separately. Tubes/beads were washed ×3 with PBS. Blocked phage was applied to coated and blocked tubes and incubated for 2 h at room temperature. Tubes/beads were washed, and bound phage was eluted with 1 mL 100 μM triethylamine. Eluted phage was incubated with TG1 *E. coli* with an OD 0.5 for 40 min. Bacteria were pelleted and plated on agar plates.

Selection of anti-B7-H3 scFvs

Selected colonies from panned libraries were grown in 96-well plates. Positive binders were identified using ELISA against immobilized recombinant B7-H3. Bound scFv-myc was detected with anti-myc (Sigma) followed by an anti-rabbit HRP (Sigma). Selected binders were cloned into scFv-Fc format in a pcDNA3.1 expression vector. 293F cells were transiently transfected with PEI (Sigma). Cells were cultured until viability dropped and supernatant was harvested.

Protein was purified on HiTrap MabSelect Protein-A Columns (Cytiva). Purified, diluted protein was used to stain cell bound B7-H3 on Jurkats and the binding analyzed using flow cytometry.

Production of B7-H3-positive Jurkat cells

A truncated B7-H3 (T-B7-H3) in an SFG γ-retroviral expression cassette was a gift from Karin Straathof (UCL). A 4Ig-B7-H3 isoform of B7-H3 was purchased (Sinobiological) and cloned into a γ-retroviral expression cassette. 4Ig-B7-H3 was digested to produce 2Ig-B7-H3. Retroviral transduction was used to stably transduce Jurkat cells with each of these three isoforms of B7-H3.

PBMC and T cell isolation

Leukapheresis cones were acquired from NHS Blood and Transplant. PBMCs were separated through ficoll centrifugation using Lymphoprep (STEMCELL Technologies). PBMCs were washed and residual red cells lysed with ACK Lysis buffer (Thermo Fisher Scientific). NK cells were depleted using magnetic CD56 depletion beads (Miltenyi Biotec) and LD depletion columns (Miltenyi Biotec).

Generation of CAR-T constructs

Geneblocks for each of the anti-B7-H3 CAR-T cells were designed and cloned into a previously described expression vector scFv-CH2-CH3-CD28-CD3ζ γ-retroviral CAR expression cassette with an RQR8 marker gene for selection/elimination based on CD34 expression,[47] using restriction sites at the 3' and 5' ends of the CAR (Thermo Fisher Scientific). These geneblocks included TE9-CD8H/Tm-CD28-CD3ζ, TC6-CD8H/Tm-CD28-CD3ζ, TF9-CD8H/Tm-CD28-CD3ζ, BF9-CD8H/Tm-CD28-CD3ζ, BH6-CD8H/Tm-CD28-CD3ζ, TE9-CD8H/Tm-4-1BB-CD3ζ, TE9-CD28H/Tm-CD28-CD3ζ, TE9-CD8H/Tm-CD28-ILR2-CD3ζ, MGA271-CD8H/Tm-CD28-CD3ζ, and 376.96-CD8H/Tm-CD28-CD3ζ.

CAR-T cell transduction

PBMCs were suspended in RPMI containing FCS and L-glutamine at a concentration of 1×10^6 cells/mL. They were activated with 0.5 μg/mL of anti-CD3 (Miltenyi Biotec) and anti-CD28 antibodies (Miltenyi Biotec). Forty-eight hours before transduction and on the day of transduction, 100 IU/mL recombinant human IL-2 (Proleukin, Novartis) was added. T cells were transduced using γ-retroviral transduction. Transduction efficiency was measured 3 days after transduction using flow cytometry by staining for CD34 (R&D). T cell populations were not corrected for transduction efficiency in functional assays.

T cell functional assays

For the 18-h co-culture assay, CAR-T cells were co-cultured with LAN-1, Kelly, K562, or no antigen stimulus in 48-well plates at an effector: target ratio of 2:1. After 18 h, supernatant was removed for ELISA and cells incubated with monensin (BioLegend). Activation markers CD69 and CD25, and the degranulation marker CD107a, were detected by flow cytometry. For the 7-day co-culture, CAR-T cells were labelled with CSFE (Thermo Fisher Scientific) co-cultured with LAN-1, Kelly, K562, or no antigen target in a 24-well plate for 6 days at an effector:target ratio of 2:1. On the 6th day, plates were centrifuged to pellet cells, 1 mL medium was removed and 1 mL added containing fresh target cells. After a further 24 h, the supernatant was removed for ELISA and the cells pelleted and the levels of exhaustion markers Tim3, Lag-3, and PD-1 and proliferation as measured by CSFE dilution were examined using flow cytometry.

To evaluate the proliferative capacity of the IL-2Rβ-modified CAR construct, CAR-T cells were labelled with CellTrace Violet (Thermo Fisher Scientific) and co-cultured with wild-type Jurkats, B7-H3-expressing Jurkats, or no target cells for 6 days at an effector:target ratio of 1:1 in 48-well plates. Cells were plated with either no cytokine, 70 ng/mL IL-15 (PeproTech), or 100 IU/mL IL-2 (Proleukin, Novartis), respectively, and were fed with fresh target cells on days 2 and 4 of co-culture. Cell proliferation and fold expansion were evaluated on the 6th day by flow cytometric analysis using Precision Count Beads (BioLegend).

For the 28-day co-culture assay, CAR-T cells were co-cultured with irradiated LAN-1, Kelly, or no target cells in 24-well plates at an effector:target ratio of 2:1. Cell medium was replenished every 2–3 days. CAR-T cells were challenged with irradiated target cells every 6 days, cultured for a further 24 h, and analyzed. Cells were pelleted and supernatant was removed every week for ELISA. CAR-T cell proliferation was measured weekly by flow cytometry using Precision Count Beads (BioLegend). The levels of cytokines IL-2 and IFN-γ were quantified using ELISA MAX Deluxe Set Human IL-2 and ELISA MAX Deluxe Set Human IFN-γ (BioLegend).

Cytotoxicity was tested using a Cr^{51} release cytotoxicity assay. Target cells were incubated with Cr^{51} for 1 h then washed and plated in 96-well plates. CAR-T cells or untransduced cells were plated at effector:target ratios of 10:1, 5:1, 2.5:1, and 1.25:1. The plates were incubated for 4 h at 37°C and then the supernatant was removed and incubated with scintillation fluid (PerkinElmer) overnight at room temperature. Cr^{51} released into the supernatant was measured using a 1450 MicroBeta TriLux (PerkinElmer).

The activity of CAR-T cells against decreasing concentrations of B7-H3 protein was measured using a plate-based assay. ELISA plates were coated in decreasing concentrations of recombinant B7-H3 and incubated overnight at 4°C. Plates were washed and CAR-T cells or untransduced cells added. Plates were incubated overnight at 37°C, cells were pelleted, and the supernatant removed for use in ELISA.

Antibodies and flow cytometry analysis

The following antibodies were used in this study: anti-B7-H3 (FM276, Miltenyi Biotech), anti-GD2 (14.G2a, BD Biosciences), human Ig (polyclonal, Thermo Fisher Scientific), anti-mouse IgG (polyclonal, R&D), anti-CD3 (UCHT1, BioLegend), anti-HisTag (J095G45, BioLegend), anti-CD34 (QBEnd10, R&D), anti-ab-TCR (IP26, BioLegend), anti-CD107a (H4A3, BioLegend), anti-cD25 (BC96, BioLegend), anti-CD69 (FN50, BioLegend), anti-Tim3 (F38-2E2, BioLegend), anti-Lag3 (11C3C65, BioLegend), anti-PD-1 (EH12.1, BD Biosciences), anti-mouse CD45 (30-F11, BioLegend), anti-human

CD45 (HI30, BioLegend), Ghost Red 780 (Tonbo Biosciences), Zombie Yellow Viability Dye (BioLegend), propidium iodide (Gibco), Cell Trace Violet (Thermo Fisher Scientific), and Precision Count Beads (BioLegend).

Cross-reactivity of TC6, TE9, and BH6 whole antibodies

TC6, TF9, and BH6 were produced as chimeric antibodies with a human IgG1 Fc domain by Evitria. Antibodies purified on protein A columns (Cytiva) and were tested in ELISA against plate bound antigen, with detection using goat anti-human IgG (H+L) (SeraCare). Cross-reactivity against mouse B7-H3 was tested using flow cytometry against the mouse cell line 3T3/NA1.

In vivo LAN-1 model

Animal protocols were approved by local institutional research committees and in accordance with UK Home Office guidelines. Male NSG mice aged between 6 and 8 weeks were supplied by UCL. All experiments were carried out under UK home office licenses project license number 15981/01, personal license number 12972. NSG mice were injected with 1×10^6 LAN-1-BFP/Luc in Geltrex (Thermo Fisher Scientific) subcutaneously into the flank. CAR-T cells (1×10^7) were injected intravenously into the tail vain at day 10. Tumor size was monitored twice a week with digital calipers. Mice were given 200 μL luciferin into the scruff and imaged using a PhotonIMAGERTM optical imaging system (Biospace Lab) weekly. When tumors reached threshold size, mice were sacrificed and blood, spleen, and tumor samples taken. Cells were disaggregated using a cell strainer and residual red blood cells removed using ACK Lysis buffer (Thermo Fisher Scientific). Cells were stained and markers analyzed using flow cytometry.

Statistical analysis

All statistical analyses were performed in GraphPad Prism v.8. Unless otherwise stated, data are expressed as mean ± range. Statistical analyses of *in vitro* assays were undertaken by one-way ANOVA with Tukey multiple comparisons, except for the Cr^{51} cytotoxicity assay where a two-way ANOVA was used. For the *in vivo* analysis, tumor size and region of interest were compared using the Kruskal-Wallis test and survival analyzed by the log-rank (Mantle-Cox) test. Unless otherwise stated the p values used in this study are as follows $^{*}p \leq 0.05$, $^{**}p \leq 0.01$, $^{***}p \leq 0.001$, $^{****}p < 0.0001$.

DATA AVAILABILITY

DNA sequences have been deposited on open science framework at the following URL: https://osf.io/n8f7g/.

SUPPLEMENTAL INFORMATION

Supplemental information can be found online at https://doi.org/10.1016/j.omto.2022.08.008.

ACKNOWLEDGMENTS

The work was supported by the following research grants: CHILDREN with CANCER UK award 15-502 to K.B. and J.A., combined grant from CHILDREN with CANCER UK + GOSHCC (W1060 to M.A.-F., T.G., J.A., and K.C.), Cancer Research UK/Stand Up to Cancer Pediatric Cancer New Discoveries Challenge (RT-6188 to A.V., C.L.-B., and J.A.), CHILDREN with CANCER UK (16-299 to J.A.), Solving Kids' Cancer (170119 to J.A.), joint grant GOSHCC and Sparks (V4219 to J.A.), Action Medical Research (GN2400 to B.F. and J.A.), Debbie Fund UCLH charity 1427 to K.C., Oliva Hodson Cancer Fund award through GOSHCC, TC-Biopharm industry PhD fellowship to G.F. and J.A., Core funding from Research into Childhood Cancer (RICC). John Anderson is supported by the NIHR Great Ormond Street Biomedical Centre. We would like to thank Karin Straathof for providing the B7H3-Fc immunogen. We would like to thank Francesca Alt for assistance with cloning of CAR constructs. Figures credited with biorender.com.

AUTHOR CONTRIBUTIONS

J.A., K.C., and K.B. designed the research proposal. K.B. and J.A. wrote the manuscript. K.B., C.L.-B., E.M.-R., M.B., A.G., A.V., M.A.-F., L.H., L.P., G.F., N.H., and B.F. performed experiments described in the manuscript.

DECLARATION OF INTERESTS

John Anderson declares founder shares in Autolus Ltd and collaborations with Roche and ALX-Oncology.

REFERENCES

1. Park, J.H., Rivière, I., Gonen, M., Wang, X., Sénéchal, B., Curran, K.J., Sauter, C., Wang, Y., Santomasso, B., Mead, E., et al. (2018). Long-term follow-up of CD19 CAR therapy in acute lymphoblastic leukemia. N. Engl. J. Med. *378*, 449–459.
2. Lee, D.W., Kochenderfer, J.N., Stetler-Stevenson, M., Cui, Y.K., Delbrook, C., Feldman, S.A., Fry, T.J., Orentas, R., Sabatino, M., Shah, N.N., et al. (2015). T cells expressing CD19 chimeric antigen receptors for acute lymphoblastic leukaemia in children and young adults: a phase 1 dose-escalation trial. Lancet *385*, 517–528.
3. Mohty, M., Gautier, J., Malard, F., Aljurf, M., Bazarbachi, A., Chabannon, C., Kharfan-Dabaja, M.A., Savani, B.N., Huang, H., Kenderian, S., et al. (2019). CD19 chimeric antigen receptor-T cells in B-cell leukemia and lymphoma: current status and perspectives. Leukemia *33*, 2767–2778.
4. Ramakrishna, S., Barsan, V., and Mackall, C. (2020). Prospects and challenges for use of CAR T cell therapies in solid tumors. Expert Opin. Biol. Ther. *20*, 503–516.
5. Stratton, M.R., Campbell, P.J., and Futreal, P.A. (2009). The cancer genome. Nature *458*, 719–724.
6. Park, J.A., and Cheung, N.K.V. (2017). Limitations and opportunities for immune checkpoint inhibitors in pediatric malignancies. Cancer Treat. Rev. *58*, 22–33.
7. Du, H., Hirabayashi, K., Ahn, S., Kren, N.P., Montgomery, S.A., Wang, X., Tiruthani, K., Mirlekar, B., Michaud, D., Greene, K., et al. (2019). Antitumor responses in the absence of toxicity in solid tumors by targeting B7-H3 via chimeric antigen receptor T cells. Cancer Cell *35*, 221–237.e8.
8. Majzner, R.G., Rietberg, S.P., Sotillo, E., Dong, R., Vachharajani, V.T., Labanieh, L., Myklebust, J.H., Kadapakkam, M., Weber, E.W., Tousley, A.M., et al. (2020). Tuning the antigen density requirement for CAR T-cell activity. Cancer Discov. *10*, 702–723.
9. Chapoval, A.I., Ni, J., Lau, J.S., Wilcox, R.A., Flies, D.B., Liu, D., Dong, H., Sica, G.L., Zhu, G., Tamada, K., and Chen, L. (2001). B7-H3: a costimulatory molecule for T cell activation and IFN-gamma production. Nat. Immunol. *2*, 269–274.
10. Wang, L., Kang, F.B., and Shan, B.E. (2014). B7-H3-mediated tumor immunology: friend or foe? Int. J. Cancer *134*, 2764–2771.
11. Maachani, U.B., Tosi, U., Pisapia, D.J., Mukherjee, S., Marnell, C.S., Voronina, J., Martinez, D., Santi, M., Dahmane, N., Zhou, Z., et al. (2020). B7–H3 as a prognostic biomarker and therapeutic target in pediatric central nervous system tumors. Transl. Oncol. *13*, 365–371.

12. Theruvath, J., Sotillo, E., Mount, C.W., Graef, C.M., Delaidelli, A., Heitzeneder, S., Labanieh, L., Dhingra, S., Leruste, A., Majzner, R.G., et al. (2020). Locoregionally administered B7-H3-targeted CAR T cells for treatment of atypical teratoid/rhabdoid tumors. Nat. Med. *26*, 712–719.

13. Modak, S., Kramer, K., Gultekin, S.H., Guo, H.F., and Cheung, N.K. (2001). Monoclonal antibody 8H9 targets a novel cell surface antigen expressed by a wide spectrum of human solid tumors. Cancer Res. *61*, 4048–4054.

14. Majzner, R.G., Theruvath, J.L., Nellan, A., Heitzeneder, S., Cui, Y., Mount, C.W., Rietberg, S.P., Linde, M.H., Xu, P., Rota, C., et al. (2019). CAR T cells targeting B7-H3, a pan-cancer antigen, demonstrate potent preclinical activity against pediatric solid tumors and brain tumors. Clin. Cancer Res. *25*, 2560–2574.

15. Steinberger, P., Majdic, O., Derdak, S.V., Pfistershammer, K., Kirchberger, S., Klauser, C., Zlabinger, G., Pickl, W.F., Stöckl, J., and Knapp, W. (2004). Molecular characterization of human 4Ig-B7-H3, a member of the B7 family with four Ig-like domains. J. Immunol. *172*, 2352–2359.

16. Clark, H.F., Gurney, A.L., Abaya, E., Baker, K., Baldwin, D., Brush, J., Chen, J., Chow, B., Chui, C., Crowley, C., et al. (2003). The secreted protein discovery initiative (SPDI), a large-scale effort to identify novel human secreted and transmembrane proteins: a bioinformatics assessment. Genome Res. *13*, 2265–2270.

17. Sun, J., Fu, F., Gu, W., Yan, R., Zhang, G., Shen, Z., Zhou, Y., Wang, H., Shen, B., and Zhang, X. (2011). Origination of new immunological functions in the costimulatory molecule B7-H3: the role of exon duplication in evolution of the immune system. PLoS One *6*, e24751.

18. Leitner, J., Klauser, C., Pickl, W.F., Stöckl, J., Majdic, O., Bardet, A.F., Kreil, D.P., Dong, C., Yamazaki, T., Zlabinger, G., et al. (2009). B7-H3 is a potent inhibitor of human T-cell activation: No evidence for B7-H3 and TREML2 interaction. Eur. J. Immunol. *39*, 1754–1764.

19. Chen, C., Shen, Y., Qu, Q.X., Chen, X.Q., Zhang, X.G., and Huang, J.A. (2013). Induced expression of B7-H3 on the lung cancer cells and macrophages suppresses T-cell mediating anti-tumor immune response. Exp. Cell Res. *319*, 96–102.

20. Hashiguchi, M., Kobori, H., Ritprajak, P., Kamimura, Y., Kozono, H., and Azuma, M. (2008). Triggering receptor expressed on myeloid cell-like transcript 2 (TLT-2) is a counter-receptor for B7-H3 and enhances T cell responses. Proc. Natl. Acad. Sci. USA *105*, 10495–10500.

21. Husain, B., Ramani, S.R., Chiang, E., Lehoux, I., Paduchuri, S., Arena, T.A., Patel, A., Wilson, B., Chan, P., Franke, Y., et al. (2019). A platform for extracellular interactome discovery identifies novel functional binding partners for the immune receptors B7-H3/CD276 and PVR/CD155. Mol. Cell. Proteomics *18*, 2310–2323.

22. Zhang, X., Fang, C., Zhang, G., Jiang, F., Wang, L., and Hou, J. (2017). Prognostic value of B7-H3 expression in patients with solid tumors: a meta-analysis. Oncotarget *8*, 93156–93167.

23. Li, Y., Yang, X., Wu, Y., Zhao, K., Ye, Z., Zhu, J., Xu, X., Zhao, X., and Xing, C. (2017). B7-H3 promotes gastric cancer cell migration and invasion. Oncotarget *8*, 71725–71735.

24. Li, D., Wang, J., Zhou, J., Zhan, S., Huang, Y., Wang, F., Zhang, Z., Zhu, D., Zhao, H., Li, D., et al. (2017). B7-H3 combats apoptosis induced by chemotherapy by delivering signals to pancreatic cancer cells. Oncotarget *8*, 74856–74868.

25. Liu, H., Tekle, C., Chen, Y.W., Kristian, A., Zhao, Y., Zhou, M., Liu, Z., Ding, Y., Wang, B., Mælandsmo, G.M., et al. (2011). B7-H3 silencing increases paclitaxel sensitivity by abrogating Jak2/Stat3 phosphorylation. Mol. Cancer Ther. *10*, 960–971.

26. Loo, D., Alderson, R.F., Chen, F.Z., Huang, L., Zhang, W., Gorlatov, S., Burke, S., Ciccarone, V., Li, H., Yang, Y., et al. (2012). Development of an Fc-enhanced anti-B7-H3 monoclonal antibody with potent antitumor activity. Clin. Cancer Res. *18*, 3834–3845.

27. Imai, K., Wilson, B.S., Bigotti, A., Natali, P.G., and Ferrone, S. (1982). A 94, 000-dalton glycoprotein expressed by human melanoma and carcinoma cells. J. Natl. Cancer Inst. *68*, 761–769.

28. Irvine, L., and Stiller, C. (2018). Childhood Cancer Statistics, England Annual Report 2018 (Public Health England).

29. Matthay, K.K., Maris, J.M., Schleiermacher, G., Nakagawara, A., Mackall, C.L., Diller, L., and Weiss, W.A. (2016). Nat. Rev. Dis. Primers *2*, 16078.

30. Straathof, K., Flutter, B., Wallace, R., Jain, N., Loka, T., Depani, S., Wright, G., Thomas, S., Cheung, G.W.-K., Gileadi, T., et al. (2020). Antitumor activity without on-target off-tumor toxicity of GD2–chimeric antigen receptor T cells in patients with neuroblastoma. Sci. Transl. Med. *12*, eabd6169.

31. Long, A.H., Haso, W.M., Shern, J.F., Wanhainen, K.M., Murgai, M., Ingaramo, M., Smith, J.P., Walker, A.J., Kohler, M.E., Venkateshwara, V.R., et al. (2015). 4-1BB costimulation ameliorates T cell exhaustion induced by tonic signaling of chimeric antigen receptors. Nat. Med. *21*, 581–590.

32. Muller, Y.D., Nguyen, D.P., Ferreira, L.M.R., Ho, P., Raffin, C., Valencia, R.V.B., Congrave-Wilson, Z., Roth, T.L., Eyquem, J., Van Gool, F., et al. (2021). The CD28-transmembrane domain mediates chimeric antigen receptor heterodimerization with CD28. Front. Immunol. *12*, 639818.

33. Kagoya, Y., Tanaka, S., Guo, T., Anczurowski, M., Wang, C.H., Saso, K., Butler, M.O., Minden, M.D., and Hirano, N. (2018). A novel chimeric antigen receptor containing a JAK-STAT signaling domain mediates superior antitumor effects. Nat. Med. *24*, 352–359.

34. Tajiri, M. (2020). Comparison of high-throughput sequencing for phage display peptide screening on two commercially available platforms. Int. J. Pept. Res. Ther. *26* (1), 523–529.

35. Noh, J., Kim, O., Jung, Y., Han, H., Kim, J.-E., Kim, S., Lee, S., Park, J., Jung, R.H., Kim, S.I., et al. (2019). High-throughput retrieval of physical DNA for NGS-identifiable clones in phage display library. mAbs *11*, 532–545.

36. Glanville, J., Zhai, W., Berka, J., Telman, D., Huerta, G., Mehta, G.R., Ni, I., Mei, L., Sundar, P.D., Day, G.M.R., et al. (2009). Precise determination of the diversity of a combinatorial antibody library gives insight into the human immunoglobulin repertoire. Proc. Natl. Acad. Sci. USA *106*, 20216–20221.

37. Persson, H., Ye, W., Wernimont, A., Adams, J.J., Koide, A., Koide, S., Lam, R., and Sidhu, S.S. (2013). CDR-H3 diversity is not required for antigen recognition by synthetic antibodies. J. Mol. Biol. *425*, 803–811.

38. Thomas, S., Straathof, K., Himoudi, N., Anderson, J., and Pule, M. (2016). An optimized GD2-targeting retroviral cassette for more potent and safer cellular therapy of neuroblastoma and other cancers. PLoS One *11*, e0152196.

39. Hombach, A.A., Schildgen, V., Heuser, C., Finnern, R., Gilham, D.E., and Abken, H. (2007). T cell activation by antibody-like immunoreceptors: the position of the binding epitope within the target molecule determines the efficiency of activation of redirected T cells. J. Immunol. *178*, 4650–4657.

40. Alabanza, L., Pegues, M., Geldres, C., Shi, V., Wiltzius, J.J.W., Sievers, S.A., Yang, S., and Kochenderfer, J.N. (2017). Function of novel anti-CD19 chimeric antigen receptors with human variable regions is affected by hinge and transmembrane domains. Mol. Ther. *25*, 2452–2465.

41. Labanieh, L., Majzner, R.G., and Mackall, C.L. (2018). Programming CAR-T cells to kill cancer. Nat. Biomed. Eng. *2*, 377–391.

42. Richards, R.M., Sotillo, E., and Majzner, R.G (2018). CAR T cell therapy for neuroblastoma. Front. Immunol. *9*, 2380.

43. Airoldi, I., Meazza, R., Croce, M., Di Carlo, E., Piazza, T., Cocco, C., D'Antuono, T., Pistoia, V., Ferrini, S., and Corrias, M.V. (2004). Low-dose interferon-γ-producing human neuroblastoma cells show reduced proliferation and delayed tumorigenicity. Br. J. Cancer. https://doi.org/10.1038/sj.bjc.6601842.

44. Pistoia, V., Morandi, F., Bianchi, G., Pezzolo, A., Prigione, I., and Raffaghello, L. (2013). Immunosuppressive microenvironment in neuroblastoma. Front. Oncol. *3*, 167.

45. Mussai, F., Egan, S., Hunter, S., Webber, H., Fisher, J., Wheat, R., McConville, C., Sbirkov, Y., Wheeler, K., Bendle, G., et al. (2015). Neuroblastoma arginase activity creates an immunosuppressive microenvironment that impairs autologous and engineered immunity. Cancer Res. *75*, 3043–3053.

46. Montironi, C., Muñoz-Pinedo, C., and Eldering, E. (2021). Hematopoietic versus solid cancers and T cell dysfunction: looking for similarities and distinctions. Cancers (Basel) *13*, 284.

47. Philip, B., Kokalaki, E., Mekkaoui, L., Thomas, S., Straathof, K., Flutter, B., Marin, V., Marafioti, T., Chakraverty, R., Linch, D., et al. (2014). A highly compact epitope-based marker/suicide gene for easier and safer T-cell therapy. Blood *124*, 1277–1287.

OMTO, Volume *26*

Supplemental information

A novel anti-B7-H3 chimeric antigen receptor from a single-chain antibody library for immunotherapy of solid cancers

Kathleen Birley, Clara Leboreiro-Babe, Enrique Miranda Rota, Magdalena Buschhaus, Artemis Gavriil, Alice Vitali, Maria Alonso-Ferrero, Lee Hopwood, Lara Parienti, Gabrielle Ferry, Barry Flutter, Nourredine Himoudi, Kerry Chester, and John Anderson

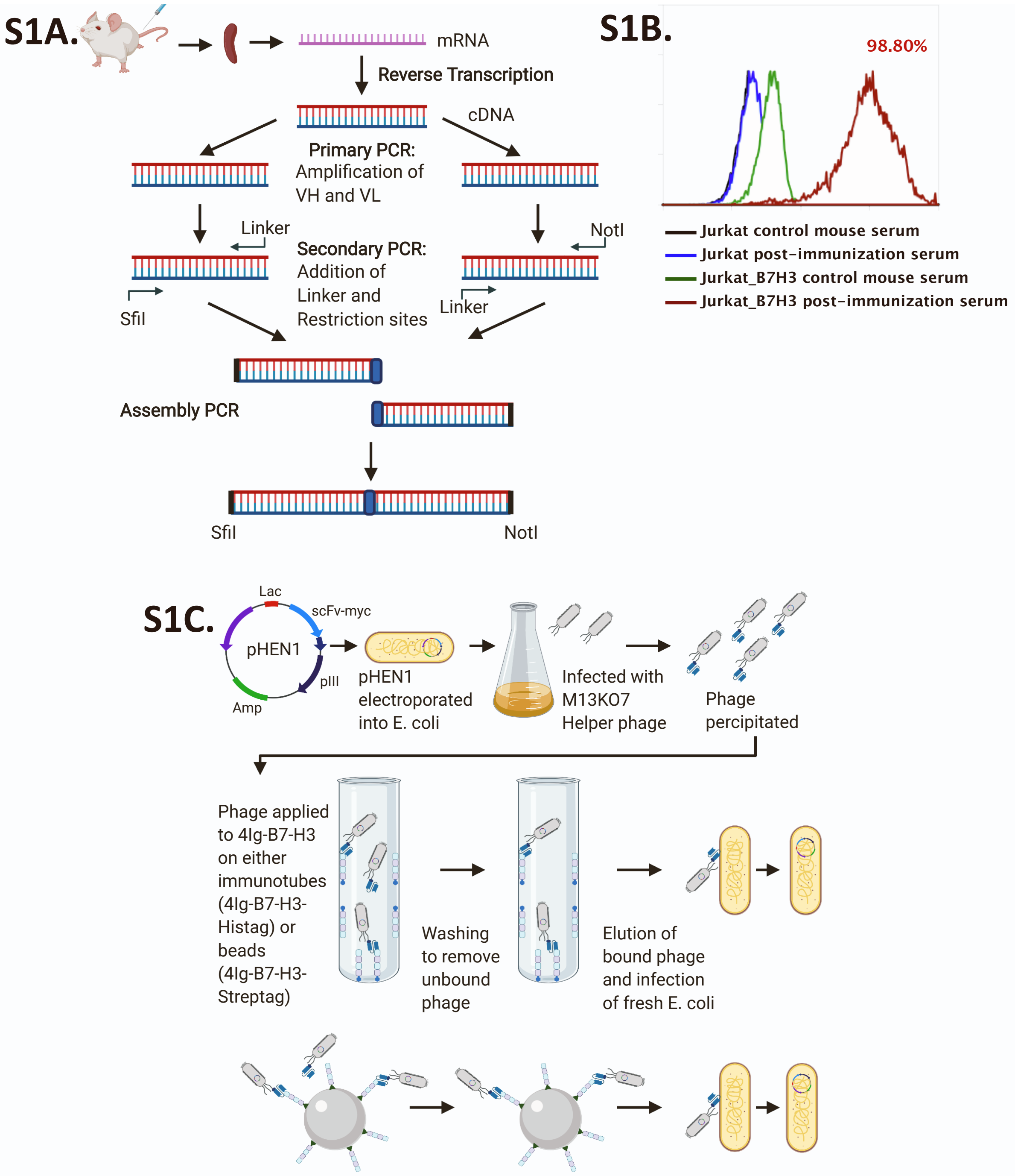

S1D.

	Immunized Library	
**Library Size**	2.06x10^7	
	**Immunotube panning**	**Bead panning**
**Library Size**	1.20x10^4	1.05x10^4

Figure S1. *Production and panning of the anti-B7-H3 library*. **A.** Production of the phage display library. Mice were immunized with a B7-H3-mouseFc fusion protein. The spleens were collected from the immunized mouse, mRNA extracted reverse transcribed into corresponding VH and VL cDNA. PCR reactions were used to add a linker and myc-tag before the scFv-myc was cloned into the pHEN phagemid. **B.** Testing of the immunized mouse serum against B7-H3 +/- Jurkat cells. **C.** The phage display library was panned against 4Ig-B7-H3 immobilized on immunotubes or magnetic beads. **D.** The size of the immunized library and the immunotube and magnetic bead panned libraries as estimated by serial dilution of phage-infected E. *coli*.

S2A.

	BB5	TB8	BH6	TE9	BF9	BB1	TA12	BF12	TA6	BC10	BA5	BF2	BG5	BG4	BD9	TC6	TF9
BB5	100.00	68.05	79.52	78.16	78.54	75.91	78.26	79.27	80.15	78.37	79.21	78.16	79.42	78.91	78.54	78.16	78.41
TB8	68.05	100.00	78.91	81.31	80.93	78.81	80.93	81.69	80.18	80.05	82.78	80.71	82.60	82.60	80.83	81.34	82.09
BH6	79.52	78.91	100.00	92.37	92.11	90.60	92.88	91.60	92.97	92.83	93.11	91.99	91.23	92.88	91.99	91.49	92.25
TE9	78.16	81.31	92.37	100.00	96.34	91.55	93.94	94.19	91.98	93.13	94.17	93.94	94.19	94.95	95.33	93.56	94.57
BF9	78.54	80.93	92.11	96.34	100.00	91.80	93.81	95.45	92.88	93.51	94.55	93.94	94.44	94.57	96.46	94.57	95.45
BB1	75.91	78.81	90.60	91.55	91.80	100.00	95.71	93.32	90.47	90.85	90.89	91.17	90.67	91.55	90.92	91.30	91.05
TA12	78.26	80.93	92.88	93.94	93.81	95.71	100.00	95.59	92.62	93.26	92.78	93.06	92.93	93.31	92.42	92.93	92.55
BF12	79.27	81.69	91.60	94.19	95.45	93.32	95.59	100.00	92.49	92.24	94.80	93.56	94.82	94.57	94.19	95.45	94.82
TA6	80.15	80.18	92.97	91.98	92.88	90.47	92.62	92.49	100.00	93.47	94.26	92.25	92.88	93.01	92.88	92.63	93.52
BC10	78.37	80.05	92.83	93.13	93.51	90.85	93.26	92.24	93.47	100.00	92.88	93.52	93.39	93.27	94.54	93.52	94.03
BA5	79.21	82.78	93.11	94.17	94.55	90.89	92.78	94.80	94.26	92.88	100.00	93.67	94.68	95.32	94.56	94.43	95.95
BF2	78.16	80.71	91.99	93.94	93.94	91.17	93.06	93.56	92.25	93.52	93.67	100.00	95.84	95.84	96.09	96.09	96.47
BG5	79.42	82.60	91.23	94.19	94.44	90.67	92.93	94.82	92.88	93.39	94.68	95.84	100.00	96.47	96.47	96.85	96.97
BG4	78.91	82.60	92.88	94.95	94.57	91.55	93.31	94.57	93.01	93.27	95.32	95.84	96.47	100.00	95.59	96.09	97.98
BD9	78.54	80.83	91.99	95.33	96.46	90.92	92.42	94.19	92.88	94.54	94.56	96.09	96.47	95.59	100.00	97.60	97.10
TC6	78.16	81.34	91.49	93.56	94.57	91.30	92.93	95.45	92.63	93.52	94.43	96.09	96.85	96.09	97.60	100.00	97.86
TF9	78.41	82.09	92.25	94.57	95.45	91.05	92.55	94.82	93.52	94.03	95.95	96.47	96.97	97.98	97.10	97.86	100.00

>60% Identical
>70% Identical
>80% Identical
>90% Identical
>95% Identical
100% Identical

S2B.

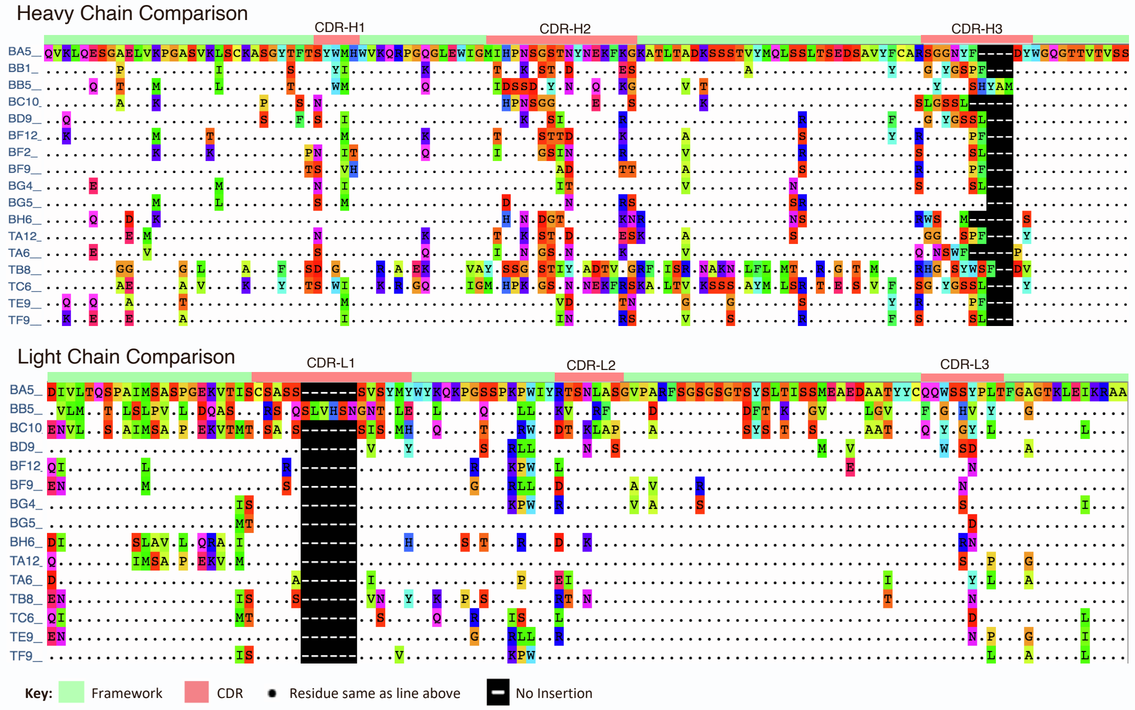

Figure S2. *The diversity of the anti-B7-H3 scFv*. **A.** A heat map showing the percentage similarities between the 17 different anti-B7-H3 scFv **B.** Alignment of the anti-B7-H3 scFv heavy and light chains arranged in alphabetical order as analyzed by Abysis (http://www.abysis.org/abysis/)

S3A.

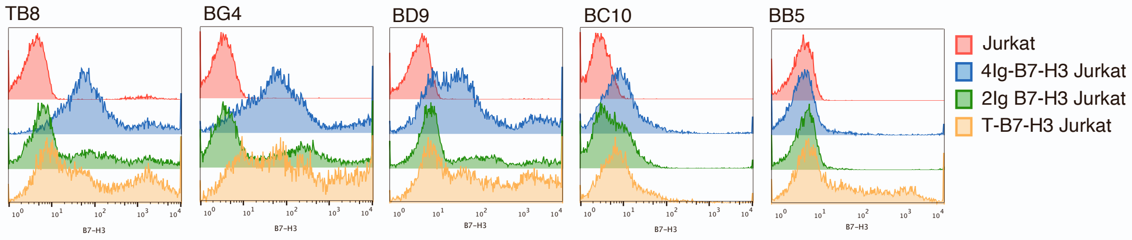

S3B.

CAR	Range	Average
TC6	61.3% - 84.1%	71.9%
TE9	58.1% - 90.5%	76.3%
TF9	34.7% - 81.9%	64.4%
BF9	72.2% - 79.6%	74.8%
BH6	40.3% - 82.1%	58.2%
GD2	23.3% - 81.3%	45.1%

S3C.

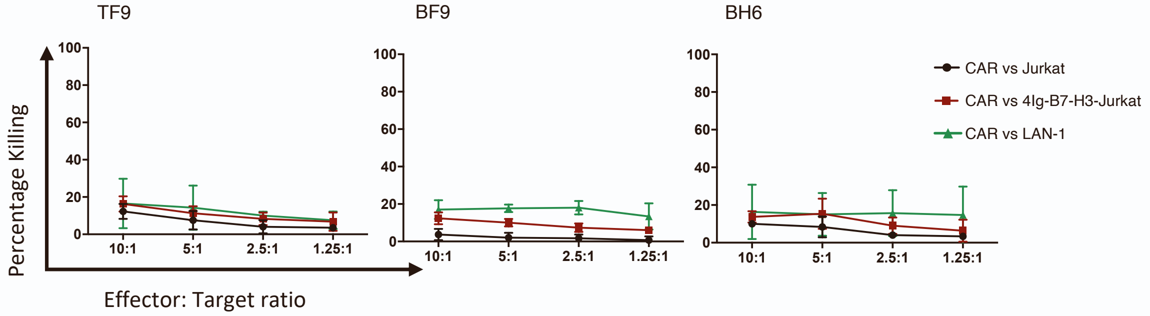

Figure S3. *The scFv not taken forward for further evaluation in CAR T cells*. **A.** The binding of scFv-Fc not taken forward against different cell-bound isoforms of B7-H3. Representative 1 of 2. **B.** The range and average transduction efficiency as determined on flow cytometry **C.** Cr^{51} cytotoxicity assay of CAR-T cells not taken forward against isogenic B7-H3 +/- cell lines and LAN-1 cells (mean and SD, TF9 n = 4, BF9 and BH6 n = 3).

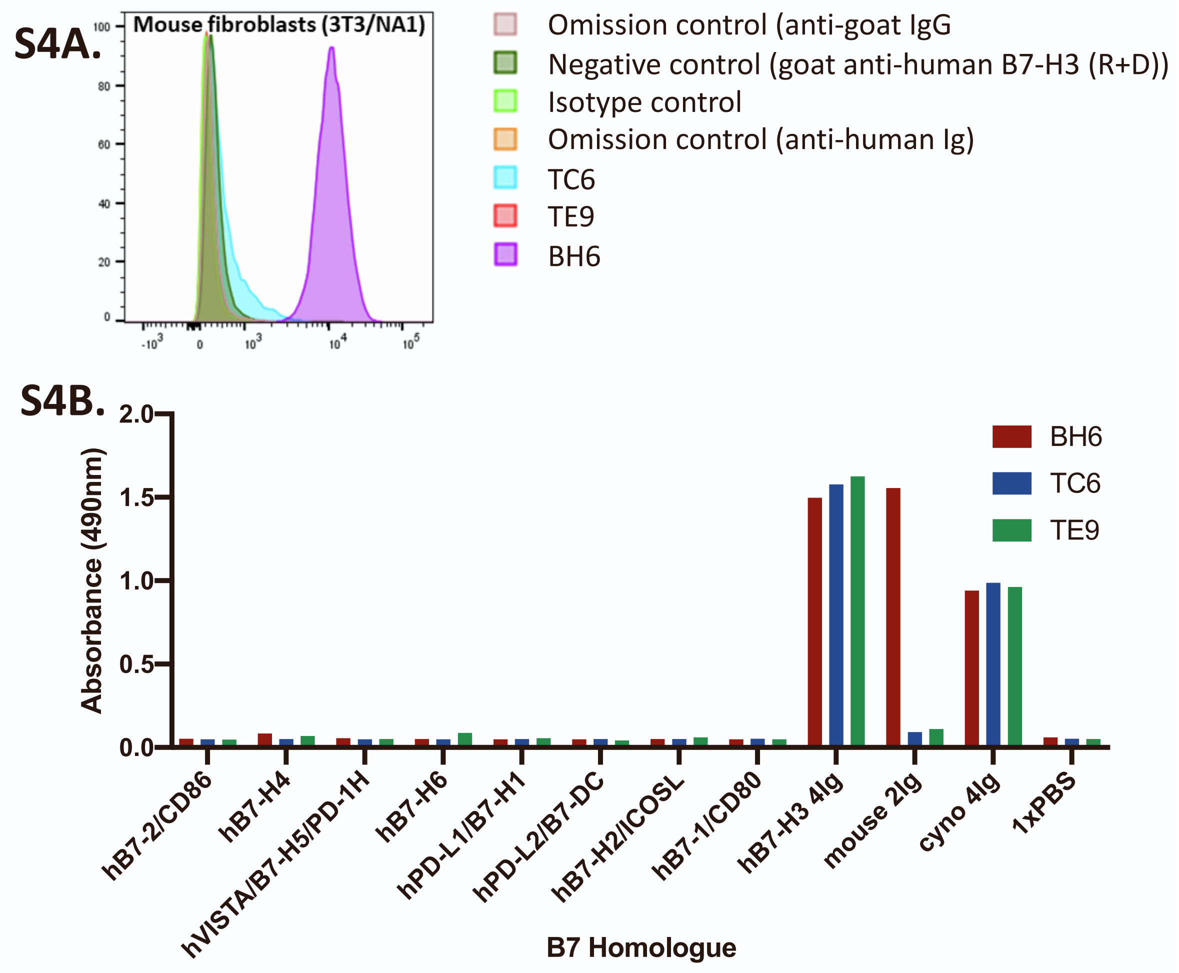

Figure S4. *Specificity of TE9, TC6 and BH6*. **A.** Staining of mouse 3T3/NA1 with TE9, TC6 and BH6 antibodies **B.** Results of ELISA showing binding of antibodies TE9, TC6 and BH6 against recombinant human B7 family proteins and non-human primate B7-H3

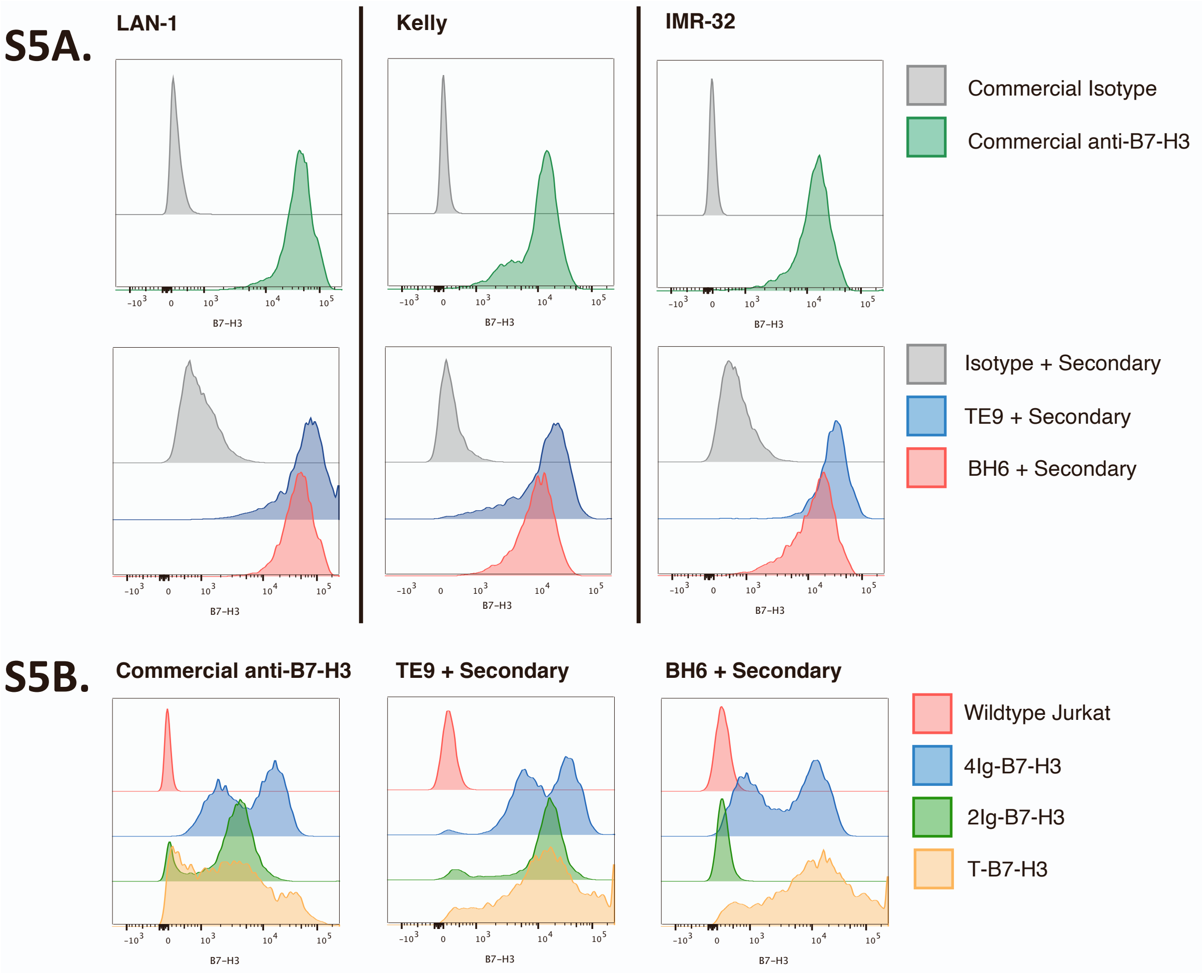

Figure S5. *Chimeric antibodies of TE9 and BH6 show a similar pattern of staining as scFv-Fc proteins and stain neuroblastoma cell lines*. **A.** Chimeric antibodies of TE9 and BH6 were used to stain 3 neuroblastoma cell lines, LAN-1, Kelly and IMR-32. The top line shows staining with a commercial directly conjugated antibody and the bottom line with the chimeric antibodies and an anti-human secondary. **B.** Commercial anti-B7-H3 antibodies and the chimeric antibodies TE9 and BH6 were used to stain the 3 B7-H3 positive Jurkat cell lines, 4Ig-B7-H3, 2Ig-B7-H3 and T-B7-H3

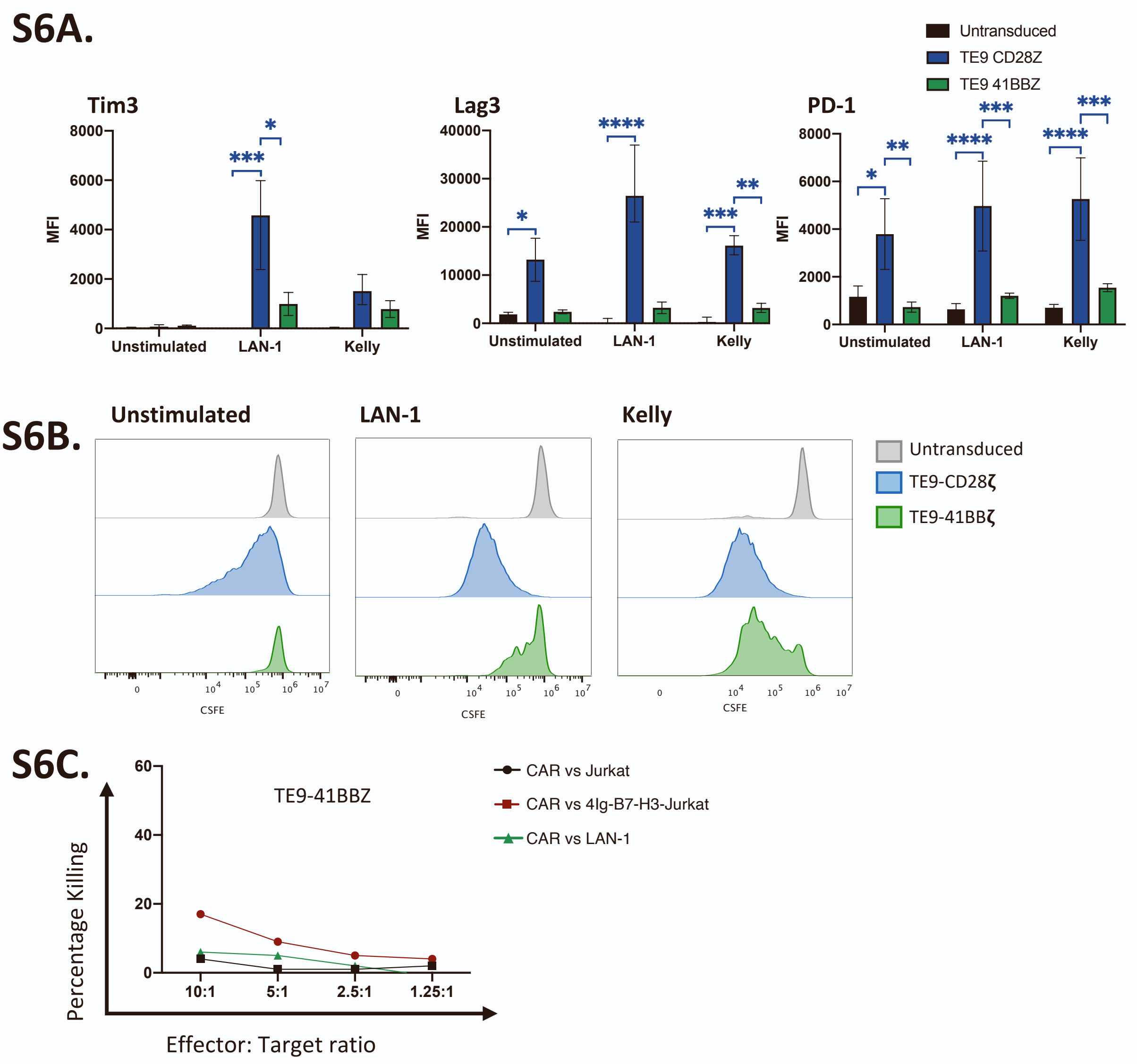

Figure S6. *TE9-28ζ expresses higher levels of Tim3, Lag3 and PD-1 following 7-day co-culture with antigen positive target cells than TE9-41BBζ.* **A.** CAR-T cells and Untransduced T-cells were cultured with LAN-1 or Kelly targets, or no antigen stimulus for 7 days then restimulated with fresh antigen targets and incubated for a further 24 hours. Cells were stained for Tim3, Lag3 and PD-1 (mean and range, n = 3, * $p \leq 0.05$; ** $p \leq 0.01$; *** $p \leq 0.001$; **** $p<0.0001$) **B.** Proliferation of TE9-CD28ζ, TE9-41BBζ and untransduced T-Cells when following 7-day co-culture and repeat stimulation assay with either LAN-1, Kelly or UT as measured using CSFE dilution, n = 1. **C.** Cytotoxicity of TE9-41BBζ against B7-H3 +/- Jurkat cells and LAN-1 measured using a chromium release assay, n = 1.

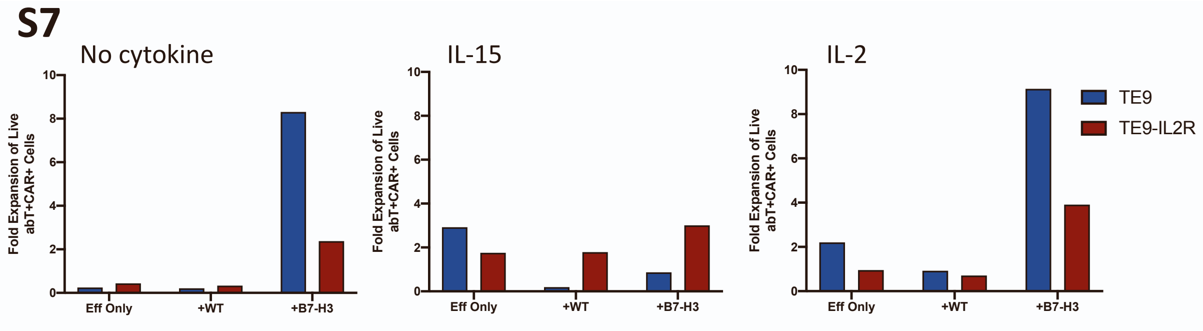

Figure S7. *TE9-28-ILR2ζ does not show proliferative superiority compared with TE9-28ζ.* **A.** CAR T cells transduced with either TE9-28ζ or TE9-28-ILR2ζ and cultured with B7-H3+/- isogenic Jurkat cell lines. Cells were cultured in the presence of IL-15, IL-2, or no cytokine stimulus. Proliferation in the form of fold change was measured on day 6 of culture using precision count beads (n = 1)

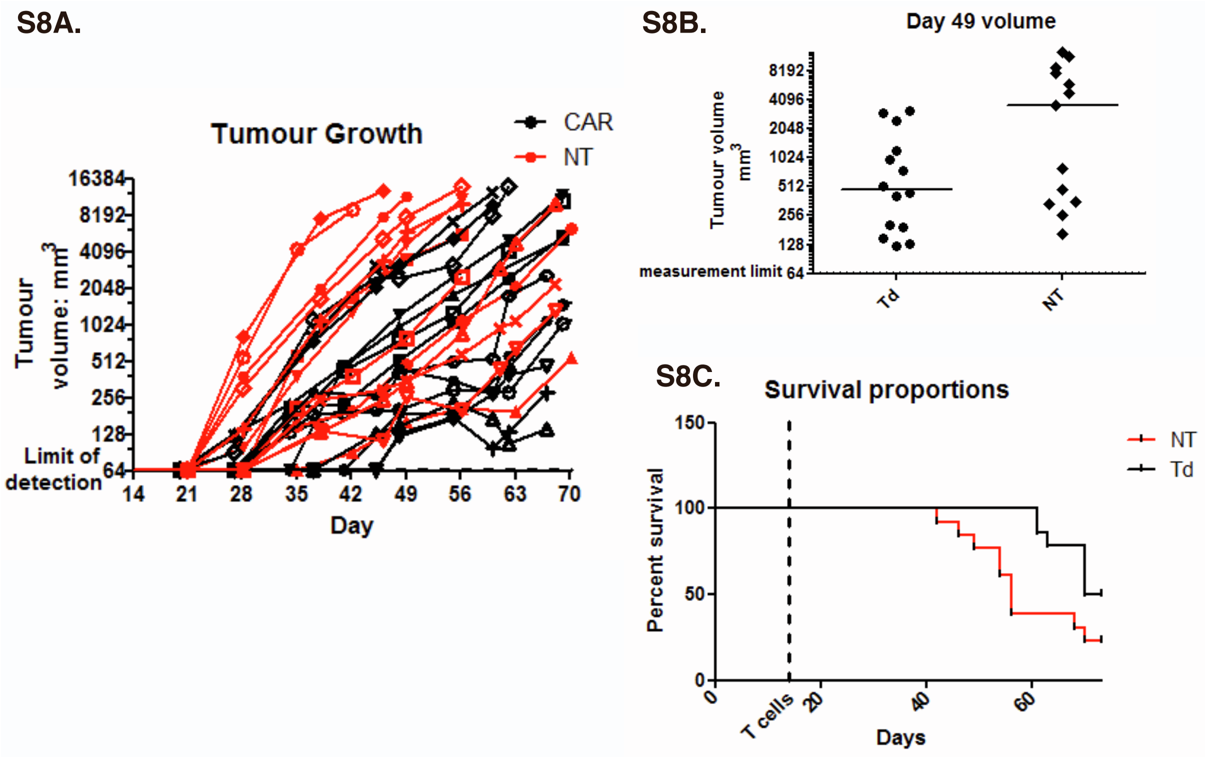

Figure S8 *Anti-GD2-CD28-CD3ζ CAR T cells do not significantly increase survival or reduce tumor size in NSG mice bearing LAN-1 tumors*. 1x10[6] LAN-1 cells in Matrigel were injected into the flank of NSG mice. When the majority of the tumors reached threshold for detection (64mm[3]) mice were treated with CAR T-cells (around day 14) (Treated Group: Td) or not treated as a control (Not Treated: NT). Mice were culled when tumors reached threshold or at the end of the experiment. **A.** The tumor growth curves for the individual tumors**. B.** The mean and individual volumes at day 49**. C** The survival curves for treatment and control mice. No significant difference was seen between treatment and control mice.

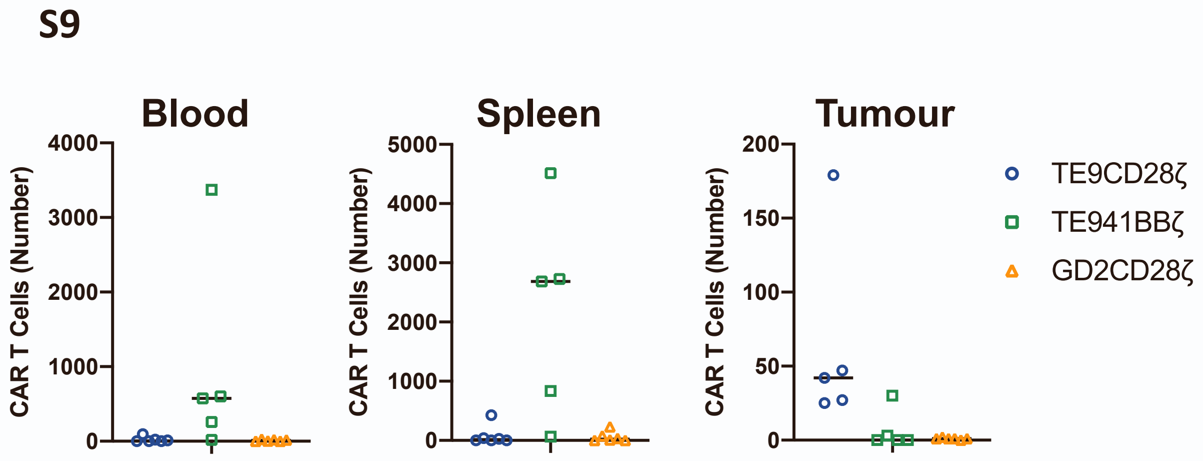

Figure S9. *TE9-28ζ CAR T Cells showed greater penetrance and survival within the tumor compared with the other treatment groups*. Blood, spleen, and tumor samples were harvested at the time of sacrifice for humane endpoints and analyzed for persistence of CAR T cells using flow cytometry. The graphs show the total number of CAR T cells detected in each sample as determined by human CD45, human CD3 and CD34 co-expression. GD2-CD28**ζ**, n = 6 for all samples, TE9-CD28**ζ** n = 6 (blood and spleen), n = 5 (tumour). TE9-41BB**ζ** n = 5 for all samples. CAR-T number is not normalized for size or volume of tissue.
